# HIV incidence among women using intramuscular depot medroxyprogesterone acetate, a copper intrauterine device, or a levonorgestrel implant for contraception: a randomised, multicentre, open-label trial

**DOI:** 10.1016/S0140-6736(19)31288-7

**Published:** 2019-07-27

**Authors:** Evidence for Contraceptive Options and HIV Outcomes (ECHO) Trial Consortium, Khatija Ahmed, Jared M Baeten, Mags Beksinska, Linda-Gail Bekker, Elizabeth A Bukusi, Deborah Donnell, Peter B Gichangi, Kate B Heller, G Justus Hofmeyr, Jessica Justman, Margaret Phiri Kasaro, James Kiarie, Cheryl Louw, Timothy D Mastro, Charles S Morrison, Nelly R Mugo, Gonasagrie Nair, Kavita Nanda, Zelda Nhlabatsi, Maricianah Onono, Thesla Palanee-Phillips, Melanie Pleaner, Helen Rees, Mandisa Singata-Madliki, Caitlin W Scoville, Raesibe Agnes Pearl Selepe, Kathleen Shears, Sydney Sibiya, Jennifer Smit, Petrus S Steyn, Jeffrey Stringer, Douglas Taylor, Katherine K Thomas, Julia D Welch

## Abstract

**Background:**

Observational and laboratory studies suggest that some hormonal contraceptive methods, particularly intramuscular depot medroxyprogesterone acetate (DMPA-IM), might increase women's susceptibility to HIV acquisition. We aimed to compare DMPA-IM, a copper intrauterine device (IUD), and a levonorgestrel (LNG) implant among African women seeking effective contraception and living in areas of high HIV incidence.

**Methods:**

We did a randomised, multicentre, open-label trial across 12 research sites in eSwatini, Kenya, South Africa, and Zambia. We included HIV-seronegative women aged 16–35 years who were seeking effective contraception, had no medical contraindications to the trial contraceptive methods, agreed to use the assigned method for 18 months, and reported not using injectable, intrauterine, or implantable contraception for the previous 6 months. Participants were randomly assigned (1:1:1) to receive an injection of 150 mg/mL DMPA-IM every 3 months, a copper IUD, or a LNG implant with random block sizes between 15 and 30, stratified by site. Participants were assigned using an online randomisation system, which was accessed for each randomisation by study staff at each site. The primary endpoint was incident HIV infection in the modified intention-to-treat population, including all randomised participants who were HIV negative at enrolment and who contributed at least one HIV test. The primary safety endpoint was any serious adverse event or any adverse event resulting in method discontinuation, until the trial exit visit at 18 months and was assessed in all enrolled and randomly assigned women. This study is registered with ClinicalTrials.gov, number NCT02550067.

**Findings:**

Between Dec 14, 2015, and Sept 12, 2017, 7830 women were enrolled and 7829 were randomly assigned to the DMPA-IM group (n=2609), the copper IUD group (n=2607), or the LNG implant group (n=2613). 7715 (99%) participants were included in the modified intention-to-treat population (2556 in the DMPA-IM group, 2571 in the copper IUD group, and 2588 in the LNG implant group), and women used their assigned method for 9567 (92%) of 10 409 woman-years of follow-up time. 397 HIV infections occurred (incidence 3·81 per 100 woman-years [95% CI 3·45–4·21]): 143 (36%; 4·19 per 100 woman-years [3·54–4·94]) in the DMPA-IM group, 138 (35%: 3·94 per 100 woman-years [3·31–4·66]) in the copper IUD group, and 116 (29%; 3·31 per 100 woman-years [2·74–3·98]) in the LNG implant group. In the modified intention-to-treat analysis, the hazard ratios for HIV acquisition were 1·04 (96% CI 0·82–1·33, p=0·72) for DMPA-IM compared with copper IUD, 1·23 (0·95–1·59, p=0·097) for DMPA-IM compared with LNG implant, and 1·18 (0·91–1·53, p=0·19) for copper IUD compared with LNG implant. 12 women died during the study: six in the DMPA-IM group, five in the copper IUD group, and one in the LNG implant group. Serious adverse events occurred in 49 (2%) of 2609 participants in the DMPA-IM group, 92 (4%) of 2607 participants in the copper IUD group, and 78 (3%) of 2613 participants in the LNG implant group. Adverse events resulting in discontinuation of the randomly assigned method occurred in 109 (4%) women in the DMPA-IM group, 218 (8%) women in the copper IUD group, and 226 (9%) women in the LNG implant group (p<0·0001 for DMPA-IM *vs* copper IUD and for DMPA-IM *vs* LNG implant). 255 pregnancies occurred: 61 (24%) in the DMPA-IM group, 116 (45%) in the copper IUD group, and 78 (31%) in the LNG implant group. 181 (71%) pregnancies occurred after discontinuation of randomly assigned method.

**Interpretation:**

We did not find a substantial difference in HIV risk among the methods evaluated, and all methods were safe and highly effective. HIV incidence was high in this population of women seeking pregnancy prevention, emphasising the need for integration of HIV prevention within contraceptive services for African women. These results support continued and increased access to these three contraceptive methods.

**Funding:**

Bill & Melinda Gates Foundation, US Agency for International Development and the President's Emergency Plan for AIDS Relief, Swedish International Development Cooperation Agency, South African Medical Research Council, and UN Population Fund. Contraceptive supplies were donated by the Government of South Africa and US Agency for International Development.

## Introduction

Women represent more than half of the 37 million people worldwide currently living with HIV, most of whom reside in sub-Saharan Africa, and more than 600 000 new infections occur each year among African women.^1^ Modern contraceptive methods are used by more than 700 million women worldwide, including more than 58 million African women.^2^ Use of these methods substantially improves maternal and child health by averting unintended pregnancy and sequelae, and it contributes to women's empowerment and to economic and social development. Unfortunately, 47% of women in Africa who do not want to become pregnant (more than 50 million women) have an unmet need for modern contraception.^3^

Injectable contraceptive use has increased substantially over the past few decades in Africa, with large increases in west Africa (eg, Mali and Sierra Leone), central Africa (eg, Chad), and east Africa (eg, Ethiopia, Kenya, and Uganda), in addition to high prevalence of use in South Africa and other countries in southern Africa.^4^ In many settings in Africa where HIV incidence is high, the intramuscular injectable progestin depot medroxyprogesterone acetate (DMPA-IM) is the predominant contraceptive used.^5^ Epidemiological, clinical, and laboratory studies have suggested that use of DMPA-IM might increase a woman's susceptibility to HIV, with meta-analyses finding a 40–50% increased risk.^6^, ^7^ However, all of these studies have important limitations, including an observational design and variable quality. Results have been inconsistent, with some studies finding no increase in HIV incidence among DMPA-IM users.^6^, ^7^ In 2017, WHO advised that women choosing injectable progestin-only contraceptive methods and at high risk of HIV should be informed about evidence suggesting heightened HIV risk but also about the uncertainty of a causal relationship.^8^ Use of other highly effective contraceptive methods, including long-acting, reversible methods such as intrauterine devices (IUDs) and hormonal implants, is rapidly increasing in Africa, but related data on HIV risk are sparse.

Injectable, intrauterine, and implantable contraceptives have been prioritised for programmatic delivery because of high contraceptive efficacy and safety. Robust evidence on the relative risks, particularly HIV susceptibility, and benefits of these contraceptive methods is important to inform women's decision making, provider counselling, and policy maker and regulatory decisions. Our primary objective was to compare HIV incidence among women using DMPA-IM, a copper IUD, or a levonorgestrel (LNG) implant.^9^ The LNG implant was chosen over the etonogestrel implant because it is more widely used in Africa, is widely used in oral contraceptive pills, and some data suggest that LNG has fewer glucocorticoid effects and is less hypo-oestrogenic than etonogestrel.^8^, ^10^ We included the copper IUD to have a highly effective non-hormonal comparator. Secondary and tertiary objectives included comparison of incidence of pregnancy, serious adverse events and adverse events leading to method discontinuation, and contraceptive method continuation by randomised method, and whether age or herpes simplex virus type 2 (HSV-2) serostatus modified the association between contraceptive method and HIV acquisition. The trial was overseen by the Evidence for Contraceptive Options and HIV Outcomes (ECHO) Trial Consortium comprising leadership from Africa, the USA, and WHO.

**Research in context**

**Evidence before this study**

Although no formal search was conducted, epidemiological studies over 30 years, conducted in various contexts, have suggested that women using some types of hormonal contraceptives, primarily intramuscular depot medroxyprogesterone acetate (DMPA-IM), might be at increased risk of HIV acquisition. Two well conducted meta-analyses, published in 2015 and 2016, comprehensively reviewed the epidemiological data and found 40–50% increased risks of HIV acquisition among women using DMPA IM compared with women not using hormonal contraception. However, the results across multiple studies were mixed, with some studies demonstrating increased HIV risk but others not showing increased HIV risk, and all studies had important limitations. Furthermore, few studies evaluated other contraceptive methods, particularly intrauterine devices (IUDs) and hormonal implants. A WHO expert panel reviewed all the available studies and recommended that further research was necessary to provide the high-quality evidence necessary for both greater scientific certainty and informing international guidelines.

**Added value of this study**

We did a randomised, multicentre, open-label trial comparing DMPA-IM, a copper IUD, and a levonorgestrel implant among African women seeking effective contraception and living in areas of high HIV incidence. This randomised trial did not find a substantial difference in HIV risk among the methods evaluated, and all methods were safe and highly effective.

**Implications of all the available evidence**

These results support continued and increased access to these three contraceptive methods, as well as expanded contraceptive choices, complemented by high-quality HIV and sexually transmitted infection prevention services.

## Methods

### Study design and participants

We did a randomised, multicentre, open-label trial at 12 research sites in eSwatini (one site), Kenya (one site), South Africa (nine sites), and Zambia (one site), which were selected for high HIV incidence and geographical distribution; a larger number of sites was chosen for South Africa given anticipated high HIV incidence and high use of DMPA-IM in that country, making the trial particularly relevant to that setting. Sites included freestanding research centres, university-affiliated research centres, and clinical sites providing reproductive health services. We included non-pregnant, HIV-seronegative women aged 16–35 years (women aged 16–17 were included where permissible by national regulations and local ethics review committee approval), who desired effective contraception, had no medical contraindications to the trial contraceptive methods, agreed to use the assigned method for 18 months, and reported not using injectable, intrauterine, or implantable contraception for the previous 6 months (appendix p 11). The only sexual behavioural eligibility criterion was being sexually active. For the complete list of inclusion and exclusion criteria see the appendix (p 11). Women were recruited from family planning or reproductive health clinics, clinics serving post-partum and post-abortion clients, other relevant clinics, and the general community by study community outreach staff.

The trial team, funders, and data and safety monitoring board (DSMB) agreed before initiation of the trial on a set of key operational performance metrics that would be reviewed by the DSMB during the trial and, if not met, would trigger re-evaluation of the trial's continuation (appendix p 12). Scientific, ethical, and community stakeholders provided input into the protocol design; global and site-specific community advisory groups provided ongoing input into trial conduct. Ethics review committees at each study site approved the study protocol (appendix p 13). The trial was implemented in accordance with Good Participatory Practice guidelines,^11^ using a range of advisory mechanisms that aligned to global standards in stakeholder engagement for clinical trials with HIV as the primary endpoint, including engagement of local community advisory boards and an ECHO-specific Global Community Advisory Group. All participants provided written informed consent, which included counselling about randomisation, each study contraceptive method, and their rights as research participants. The protocol is available online.

### Randomisation and masking

At enrolment, women were randomly assigned (1:1:1) to the DMPA-IM group, copper IUD group, or LNG implant group, with random block sizes between 15 and 30, stratified by site. Participants were assigned using an online randomisation system (Randomize.net; Ottawa, ON, Canada), which was accessed for each randomisation by study staff at each site. The randomisation sequence was generated by the trial's unmasked statisticians, who had no direct involvement otherwise with participants or trial sites. Because of the difficulty of masking clinicians and participants to contraceptive method, the study was open label; however, all personnel involved in HIV and pregnancy endpoint testing and adjudication were masked to group assignments. Laboratory testing was done without access to randomised group; review committees were provided with data listings without information on assignment of randomised group, contraceptive method-related data, or comment fields that could otherwise identify the randomisation assignment.

### Procedures

Participants received an injection of 150 mg/mL DMPA-IM (Depo Provera; Pfizer, Puurs, Belgium), which was provided on site at enrolment and then every 3 months until the final follow-up visit at 18 months after enrolment, a copper IUD (Optima TCu380A; Injeflex, Sao Paolo, Brazil) at enrolment, or a LNG implant (Jadelle; Bayer, Turku, Finland) at enrolment. Placement was confirmed for the LNG implant at every visit and for the copper IUD at 1 month, the final visit, and when clinically indicated. Women returned for scheduled follow-up visits at 1 month after enrolment to address initial contraceptive side-effects and every 3 months to 18 months for visits that included HIV serological testing, contraceptive counselling, and safety monitoring (appendix pp 14–17). Behavioural assessment was done at 3-month visits with standardised questionnaires in face-to-face interviews. At baseline, we tested for sexually transmitted infections (STIs; *Chlamydia trachomatis, Neisseria gonorrhoeae*, and HSV-2) and provided treatment for curable STIs using both syndromic and aetiological diagnoses. During follow-up, we provided syndromic STI management. We tested for pregnancy at enrolment, the final study visit, and when clinically indicated; women who became pregnant continued trial follow-up and were referred for further management. Women were asked about adverse events at every visit, including serious adverse events; we included hospital admissions due to pregnancy and delivery among serious adverse events. Women were counselled that they could at any time choose to discontinue the method to which they were randomly assigned and instead choose another trial method, a contraceptive method not being assessed in this trial, or no method; women who discontinued their randomly assigned method were retained in the trial. Building off various sources,^12^, ^13^, ^14^, ^15^, ^16^ we developed trial-specific contraceptive method-related counselling materials, which included the informed consent document, method-specific information sheets, a pre-randomisation flip chart, and a post-randomisation flip chart.

At every visit, participants received a comprehensive package of HIV prevention services, including HIV risk reduction counselling, participant and partner HIV and STI testing and management, condoms, and, as it became a part of national standard of prevention, pre-exposure prophylaxis (PrEP). Counselling messages related to HIV risk, including PrEP and condom use, were designed and implemented consistently across the three groups throughout the trial. Women who acquired HIV were linked to HIV care and treatment. In March, 2017, when WHO released guidance related to the use of progestin-only contraceptives by women at high risk of HIV infection, and the WHO Medical Eligibility Criteria for DMPA-IM changed from a category 1 (“a condition for which there is no restriction for the use of the contraceptive method”) to a category 2 (“a condition where the advantages of using the method generally outweigh the theoretical or proven risks”),^17^ all participants were provided with this updated information across all three groups. Site teams consistently counselled participants that none of the three contraceptive methods being used in the study provided protection against HIV or other STIs and advised women to always use condoms in addition to their contraceptive method. The study team made concerted efforts to not provide additional or differential information or counselling to women in the DMPA-IM group.

After enrolment was completed, we tested baseline serum samples from a randomly selected subset of the trial population (60 per site, 20 from each group) for medroxyprogesterone acetate using a validated, high-performance liquid chromatography–heated electrospray ionisation–tandem triple quadrupole mass spectrometry assay^18^ to understand the frequency of DMPA-IM use before randomisation (concentrations of more than 0·4 ng/mL were used to define likely use within the previous 6 months [appendix pp 25–26]) and to explore the accuracy of self-report for the trial eligibility exclusion criterion for use during that same time period.

### Outcomes

The primary endpoint was incident HIV infection, identified using a standard seroconversion algorithm (appendix p 27), occurring after enrolment. For women testing HIV seropositive, we assessed archived plasma samples from the enrolment visit using HIV RNA PCR and excluded those with detectable HIV RNA. Secondary outcomes were pregnancy (appendix p 28), serious adverse events, adverse events resulting in method discontinuation, and method continuation. The primary safety endpoint of the study was defined as any serious adverse event or any adverse event resulting in method discontinuation, until the trial exit visit at 18 months.

### Statistical analysis

The trial was designed with 80% power to detect a 50% increase in the hazard of HIV for each contraceptive method compared with each of the others (ie, DMPA-IM *vs* copper IUD, DMPA-IM *vs* LNG implant, and copper IUD *vs* LNG implant). We chose a 50% increase in HIV risk on the basis of formative work with stakeholders to determine a meaningful difference that would inform policy change.^9^ The type I error was chosen to control the family-wise error rate for the three HIV endpoint comparisons at 0·10; thus, each of the three comparisons was planned to be assessed with a two-sided type I error rate of 0·04 (and corresponding 96% CIs). We assumed an underlying HIV incidence of 3·5 per 100 woman-years, up to 18 months of follow-up per participant, 10% loss to follow-up, and a 10% dilution of treatment effect due to method discontinuation. Under these assumptions, a minimum of 250 HIV acquisition events per pairwise comparison was required, and a total sample size of 7800 was planned.

The primary outcome was assessed in the modified intention-to-treat population (including only women who tested negative for HIV at enrolment and those who contributed a HIV test at follow-up), using Cox proportional hazards regression, stratified by site, with Kaplan-Meier plots of cumulative incidence and prespecified subgroup analyses including age and HSV-2 serostatus. Prespecified subgroup analyses tested effect modification using interaction terms between subgroup and randomised group in the regression model. Follow-up was calculated on the basis of days since randomisation and was censored at the last visit with HIV status assessed. HIV seroconverters were censored at the time of first HIV-positive visit. We also did preplanned analyses restricted to continuous use of randomised method, censoring upon first discontinuation of the assigned contraceptive method. The definitions of continuous use were predefined. During follow-up, for DMPA-IM, time off method occurred beginning at 17 weeks since the last injection. For the copper IUD, time off method began immediately upon removal or expulsion, unless the IUD was replaced within 28 days, in which case no time off method was counted. For the LNG implant, time off method began immediately upon removal or expulsion unless the method was reinserted the same day. These definitions were selected on the basis of a combination of knowledge about contraceptive effectiveness and logistical considerations: the DMPA-IM period was based on WHO guidance for reinjection intervals including a window period,^15^ the copper IUD period was chosen to allow sufficient time to arrange travel to the clinic and also reflected a pragmatic approach since an IUD requires clinician presence to reinsert, and no time allowance was necessary for the LNG implant, which can only be removed in the clinic. We did continuous use analyses using both non-causal methods and causal methods. Causal analysis methods^19^ were used to estimate hazard ratios (HRs) during continuous use, with inverse probability weighting for discontinuation of randomly assigned method, adjusted for baseline covariates (younger than 25 years, having living children, living with husband or primary partner, vaginal sex without a condom, more than one sex partner, and new sex partner) and time-varying covariates (vaginal sex without a condom, more than one sex partner, and new sex partner in the previous 3 months). All enrolled and randomly assigned women were included in the safety analyses. Analyses were done with SAS, version 9.4, and R, version 3.4.1. All p values are two-sided, with a significance level of 0·05, except for the primary outcome, which used a significance level of 0·04. The DSMB met before trial initiation and approximately every 6 months to review study progress, with scheduled interim reviews at a quarter, a half, and three-quarters of estimated total follow-up.

This study is registered with ClinicalTrials.gov, number NCT02550067.

### Role of the funding source

The study funders and manufacturers of the trial contraceptive methods were not involved in the execution of the trial, interpretation of results, or decision to submit for publication. The authors designed the study, gathered the data, performed all analyses, vouch for the data completeness, prepared the manuscript, and were responsible for the decision to submit for publication.

## Results

**Figure 1:**
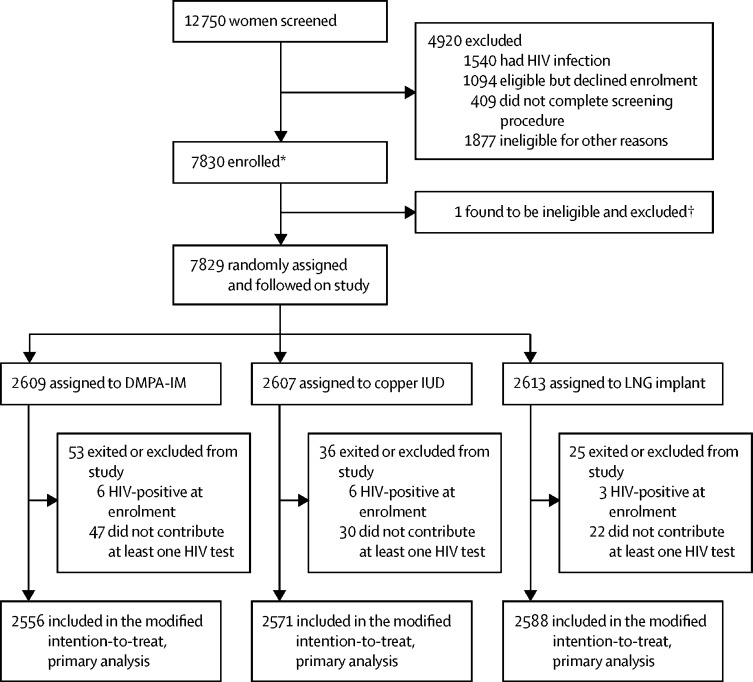
Trial profile DMPA-IM=intramuscular depot medroxyprogesterone acetate. IUD=intrauterine device. LNG=levonorgestrel. *Per-site enrolment numbers are detailed in the appendix (p 19). †One enrollee (from South Africa) was discovered 2 days after randomisation to have been younger than 18 years but had entered the trial without parental or guardian consent and was immediately exited.

Between Dec 14, 2015, and Sept 12, 2017, we enrolled 7830 women and randomly assigned 7829 women: 2609 were randomly assigned to the DMPA-IM group, 2607 to the copper IUD group, and 2613 to the LNG implant group (figure 1, appendix pp 18–19, 29). 2556 women in the DMPA-IM group, 2571 women in the copper IUD group, and 2588 women in the LNG implant group were included in the modified intention-to-treat, primary analysis. Baseline characteristics were similar among groups (table 1). Median age was 23 years (IQR 20–26), 4948 (63%) of 7829 women were younger than 25 years of age, and 64 (1%) women were aged 16–17 years. 6320 (81%) women were unmarried and 2296 (29%) were living with a partner. 6367 (81%) had been pregnant at least once. 3781 (48%) women reported no condom use with the last sexual act and 536 (7%) reported more than one sex partner in the 3 months before enrolment. STIs were common: 1420 (18%) had *C trachomatis*, 368 (5%) had *N gonorrhoeae*, and 2988 (38%) had HSV-2. Medroxyprogesterone acetate was detected at concentration of more than 0·4 ng/mL in 84 (13%) of 660 enrolment serum samples: 25 (11%) of 220 in the DMPA-IM group, 30 (14%) of 220 in the copper IUD group, and 29 (13%) of 220 in the LNG implant group.

**Table 1:** Baseline characteristics

		**DMPA-IM group (n=2609)**	**Copper IUD group (n=2607)**	**LNG implant group (n=2613)**
Age, years				
	16–17	17 (1%)	26 (1%)	21 (1%)
	18–20	695 (27%)	684 (26%)	683 (26%)
	21–24	953 (37%)	913 (35%)	956 (37%)
	25–30	719 (28%)	752 (29%)	735 (28%)
	31–35	225 (9%)	232 (9%)	218 (8%)
Marital status				
	Never married	2087 (80%)	2090 (80%)	2085 (80%)
	Married	502 (19%)	504 (19%)	503 (19%)
	Previously married	20 (1%)	13 (<1%)	25 (1%)
Lives with partner		763 (29%)	773 (30%)	760 (29%)
Education				
	No schooling	16 (1%)	12 (<1%)	21 (1%)
	Primary school	216 (8%)	247 (9%)	260 (10%)
	Secondary school	1967 (75%)	1930 (74%)	1918 (73%)
	Post secondary school	410 (16%)	418 (16%)	414 (16%)
Any previous pregnancy		2100 (80%)	2121 (81%)	2146 (82%)
Body-mass index >30 kg/m^2^		649 (25%)	663 (25%)	700 (27%)
Sexual behaviours (in past 3 months)				
	More than one sex partner	173 (7%)	198 (8%)	165 (6%)
	New sex partner	122 (5%)	131 (5%)	106 (4%)
	Number of vaginal sex acts	9 (4–20)	9 (4–20)	8 (4–20)
	Any unprotected sex	1890 (72%)	1907 (73%)	1904 (73%)
	No condom last vaginal sex	1230 (47%)	1277 (49%)	1274 (49%)
	Sex for money or gifts	32 (1%)	30 (1%)	27 (1%)
Sexually transmitted infections				
	*Chlamydia trachomatis*	454 (17%)	486 (19%)	480 (18%)
	*Neisseria gonorrhoeae*	117 (4%)	124 (5%)	127 (5%)
HSV-2*				
	Negative	1297 (50%)	1290 (49%)	1337 (51%)
	Indeterminate	290 (11%)	277 (11%)	280 (11%)
	Positive	1001 (38%)	1020 (39%)	967 (37%)

Data are n (%) or median (IQR). DMPA-IM=intramuscular depot medroxyprogesterone acetate. IUD=intrauterine device. HSV-2=herpes simplex virus type 2. LNG=levonorgestrel.

* A HSV-2 enzyme immunoassay index value of less than 0·90 was classified as negative, 0·90–3·50 as indeterminate, and more than 3·50 as positive.

Follow-up concluded on Oct 31, 2018. Follow-up was for up to 18 months, with the later-enrolling participants contributing 12–15 months of follow-up. Retention in the study was defined as contributing an HIV test result, and women who withdrew early from the study were counted as missing all subsequent visits. More than 91% of participants attended each scheduled visit to the end of follow-up in each group (appendix p 29), with 7715 (99%) participants completing at least one post-randomisation HIV test and 10 409 woman-years of follow-up accrued for assessment of HIV incidence. 7324 (94%) of 7829 women completed their final, scheduled visit or acquired HIV before their final visit. Participants attended 48 458 (94%) of 51 756 scheduled study visits. HIV testing was expected but not performed at 41 (<1%) follow-up visits.

**Figure 2:**
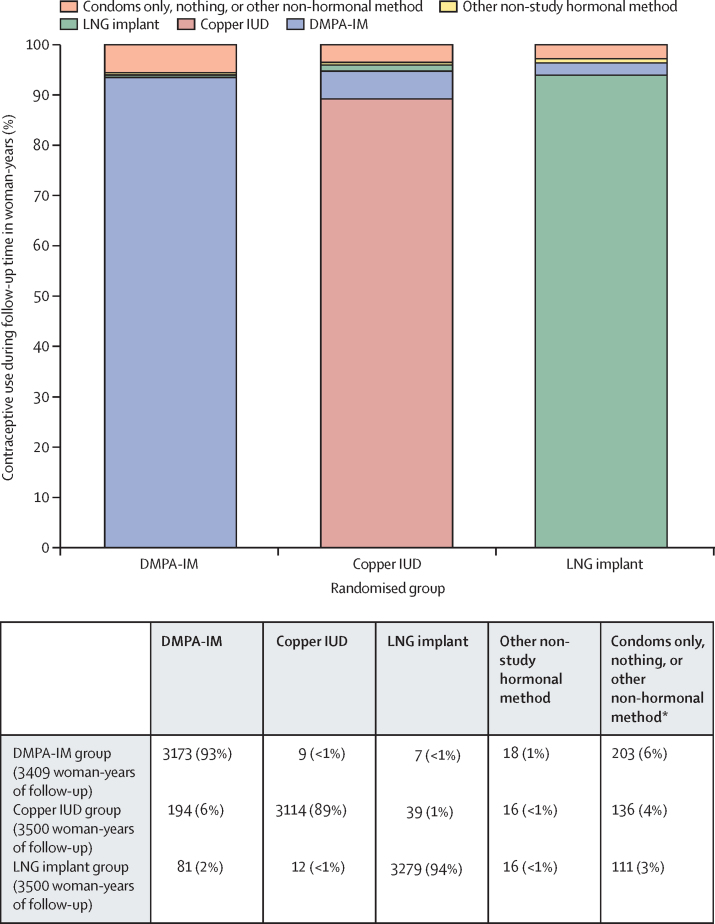
Contraceptive use by group DMPA-IM=intramuscular depot medroxyprogesterone acetate. IUD=intrauterine device. LNG=levonorgestrel. *Other non-hormonal methods accounted for less than 0·1% of contraceptive use during follow-up.

7785 (99%) women accepted their randomly assigned method at enrolment. Of 44 who initially declined their randomly assigned method, none had been assigned to the DMPA-IM group (n=2609), 36 (1%) of 2607 to the copper IUD group (five later received, four within 28 days of enrolment), and eight (<1%) of 2613 to the LNG implant group (three later received, all within 28 days of enrolment). An additional 148 did not receive their randomly assigned method on the day of enrolment for clinical reasons: three (<1%; two later received) in the DMPA-IM group, 143 in the copper IUD group (5%; 95 later received, 85 within 28 days of enrolment), and two in the LNG implant group (<1%; one later received, within 28 days of enrolment). Women used their randomly assigned method for 9567 (92%) of 10 409 woman-years: 3173 (93%) of 3409 woman-years in the DMPA-IM group, 3114 (89%) of 3500 woman-years in the copper IUD group, and 3279 (94%) of 3500 woman-years in the LNG implant group (figure 2). 14 639 (99%) of 14 760 DMPA-IM injections among women randomly assigned to DMPA-IM were provided on site. When women switched among the trial methods, the most commonly chosen method was DMPA-IM.

PrEP became national standard of care relatively late into the study; 622 women reported using PrEP (188 [30%] in the DMPA-IM group, 216 [35%] in the copper IUD group, and 218 [35%] in the LNG implant group). The median duration of use was 85 days (IQR 39–96) before study exit, and 195 woman-years (2%) of the total 10 409 woman-years of follow-up were contributed after PrEP initiation by women who started PrEP.

**Figure 3:**
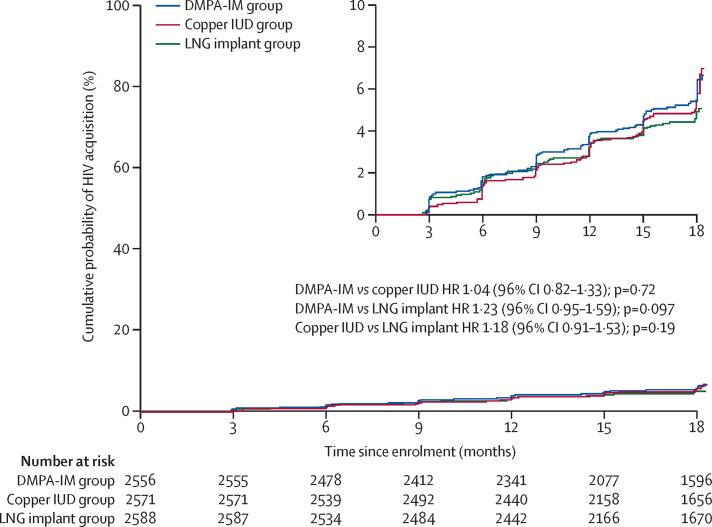
Kaplan-Meier curves for the primary, modified intention-to-treat analysis DMPA-IM=intramuscular depot medroxyprogesterone acetate. HR=hazard ratio. IUD=intrauterine device. LNG=levonorgestrel.

397 incident HIV infections were observed: 143 (36%) in the DMPA-IM group, 138 (35%) in the copper IUD group, and 116 (29%) in the LNG implant group (figure 3; table 2). Overall HIV incidence was 3·81 per 100 woman-years (95% CI 3·45–4·21): 4·19 per 100 woman-years (3·54–4·94) in the DMPA-IM group, 3·94 per 100 woman-years (3·31–4·66) in the copper IUD group, and 3·31 per 100 woman-years (2·74–3·98) in the LNG implant group. For the primary, modified intention-to-treat analysis, the HRs for HIV acquisition were 1·04 (96% CI 0·82–1·33, p=0·72) for DMPA-IM compared with copper IUD, 1·23 (0·95–1·59, p=0·097) for DMPA-IM compared with LNG implant, and 1·18 (0·91–1·53, p=0·19) for copper IUD compared with LNG implant. Although age younger than 25 years was associated with higher HIV incidence than age 25 years or older and HSV-2-seropositive status was associated with higher HIV incidence than HSV-2-seronegative status, age and HSV-2 status did not modify the association between contraceptive method and HIV acquisition; additional prespecified subgroup analyses also found no substantial differences (appendix p 20). 345 (87%) incident HIV infections occurred at the sites in South Africa; post-hoc analyses limited to the South Africa sites found similar results to the overall findings (appendix p 21).

**Table 2:** HIV incidence

		**DMPA-IM group**		**Copper IUD group**		**LNG implant group**		**DMPA-IM *vs* copper IUD**		**DMPA-IM *vs* LNG implant**		**Copper IUD *vs* LNG implant**	
		Events/participants	HIV incidence per 100 woman-years (95% CI)	Events/participants	HIV incidence per 100 woman-years (95% CI)	Events/participants	HIV incidence per 100 woman-years (95% CI)	HR (96% CI)	p value	HR (96% CI)	p value	HR (96% CI)	p value
Modified intention-to-treat analysis		143/2556	4·19 (3·54–4·94)	138/2571	3·94 (3·31–4·66)	116/2588	3·31 (2·74–3·98)	1·04 (0·82–1·33)	0·72	1·23 (0·95–1·59)	0·097	1·18 (0·91–1·53)	0·19
Continuous use analysis using causal methods		113/2392	4·19 (3·45–5·04)	121/2391	3·99 (3·31–4·77)	109/2534	3·38 (2·78–4·08)	1·10 (0·84–1·44)	0·49	1·29 (0·98–1·71)	0·060	1·18 (0·90–1·55)	0·22
Age, years		..	..	..	..	..	..	..	0·56*	..	0·63*	..	0·30*
	<25	101/1630	4·66 (3·80–5·67)	100/1599	4·61 (3·75–5·61)	78/1641	3·52 (2·78–4·39)	0·99 (0·75–1·31)	..	1·28 (0·95–1·73)	..	1·29 (0·96–1·74)	..
	≥25	42/926	3·38 (2·44–4·57)	38/972	2·85 (2·02–3·92)	38/947	2·96 (2·10–4·07)	1·16 (0·75–1·80)	..	1·13 (0·73–1·75)	..	0·97 (0·62–1·52)	..
HSV-2†		..	..	..	..	..	..	..	0·42*	..	0·40*	..	0·96*
	Negative	55/1270	3·23 (2·44–4·21)	59/1269	3·40 (2·59–4·38)	54/1326	3·01 (2·26–3·93)	0·96 (0·66–1·38)	..	1·08 (0·74–1·58)	..	1·13 (0·78–1·64)	..
	Positive	70/980	5·37 (4·18–6·78)	60/1009	4·40 (3·36–5·67)	50/956	3·84 (2·85–5·07)	1·18 (0·83–1·67)	..	1·36 (0·94–1·95)	..	1·15 (0·79–1·68)	..

DMPA-IM=intramuscular depot medroxyprogesterone acetate. IUD=intrauterine device. HR=hazard ratio. HSV-2=herpes simplex virus type 2. LNG=levonorgestrel.

* p value is for whether HR differs by subgroup. Subgroup-specific HRs are provided with 95% CIs.

† A HSV-2 enzyme immunoassay index value of less than 0·90 was classified as negative, 0·90–3·50 as indeterminate, and more than 3·50 as positive. Women with indeterminate HSV-2 results were excluded from the HSV-2 subgroup analyses.

Analyses restricted to continuous use of randomised method included 8950 woman-years (86%) of total follow-up time (2698 [79%] of 3409 woman-years for DMPA-IM, 3029 [87%] of 3500 woman-years for the copper IUD, and 3222 [92%] of 3500 woman-years for the LNG implant), and 343 (86%) of 397 incident HIV infections (appendix p 22). Using causal methods and adjusting for baseline and time-varying covariates, HRs for HIV acquisition were 1·10 (96% CI 0·84–1·44, p=0·49) for DMPA-IM compared with copper IUD, 1·29 (0·98–1·71, p=0·060) for DMPA-IM compared with LNG implant, and 1·18 (0·90–1·55, p=0·22) for copper IUD compared with LNG implant.

Across the three contraceptive method groups, self-reported coital frequency was similar over the course of follow-up (appendix p 23). Women in the DMPA-IM group less frequently reported condomless sex and multiple partners than women in the other two groups, and both DMPA-IM and LNG implant users less frequently reported new partners and sex during menses than users of the copper IUD, although absolute differences were small.

12 women died during the study: six of these women were in the DMPA-IM group (one hepatitis due to herbal medication, one due to lower respiratory tract infection, one due to sudden death, and three due to trauma [one road traffic accident, one stabbing, and one polytrauma]), five were in the copper IUD group (one due to chest pain, one due to pneumonia, one due to uterine malignancy, and two due to trauma [one road traffic accident and one polytrauma due to a gas explosion]), and one in the LNG implant group (due to superior vena cava obstruction). Serious adverse events occurred in 219 (3%) of 7829 participants: 49 (2%) of 2609 participants in the DMPA-IM group, 92 (4%) of 2607 participants in the copper IUD group, and 78 (3%) of 2613 participants in the LNG implant group (table 3). 35 (14%) of the 250 serious adverse events (none in the DMPA-IM group, 23 [22%] of 104 in the copper IUD group, and 12 [13%] of 90 in the LNG implant group) were considered to be related to the contraceptive methods. 553 (7%) of 7829 participants had an adverse event resulting in discontinuation of the randomly assigned method: 109 (4%) in the DMPA-IM group, 218 (8%) in the copper IUD group, and 226 (9%) in the LNG implant group (p<0·0001 for DMPA-IM *vs* copper IUD and for DMPA-IM *vs* LNG implant). Social harms related to trial participation occurred in 89 (1%) of participants: 25 (1%) in the DMPA-IM group, 29 (1%) in the copper IUD group, and 35 (1%) in the LNG implant group.

**Table 3:** Adverse events

		**DMPA-IM group (n=2609)**	**Copper IUD group (n=2607)**	**LNG implant group (n=2613)**	p value*		
					DMPA-IM *vs* copper IUD	DMPA-IM *vs* LNG implant	Copper IUD *vs* LNG implant
Death		6 (<1%)	5 (<1%)	1 (<1%)	0·97	0·21	0·42
Any serious adverse event		49 (2%)	92 (4%)	78 (3%)	0·00023	0·012	0·28
Any non-pregnancy-related serious adverse event		37 (1%)	71 (3%)	66 (3%)	0·00091	0·0051	0·67
Any adverse event resulting in discontinuation of randomly assigned method		109 (4%)	218 (8%)	226 (9%)	<0·0001	<0·0001	0·73
Most frequent adverse events resulting in discontinuation of randomly assigned method†							
	Menorrhagia	28 (1%)	57 (2%)	45 (2%)	0·0015	0·059	0·23
	Pelvic pain	2 (<1%)	53 (2%)	3 (<1%)	<0·0001	1·0	<0·0001
	Dysfunctional uterine bleeding	4 (<1%)	28 (1%)	17 (1%)	<0·0001	0·0071	0·10
	Headache	7 (<1%)	1 (<1%)	37 (1%)	0·070	<0·0001	<0·0001
	Weight increase	19 (1%)	0	7 (<1%)	<0·0001	0·019	0·016
	Abnormal loss of weight	3 (<1%)	3 (<1%)	18 (1%)	1·0	0·0015	0·0015
	Dysmenorrhoea	2 (<1%)	19 (1%)	1 (<1%)	0·00012	0·62	<0·0001

Data are n (%) unless otherwise stated. DMPA-IM=intramuscular depot medroxyprogesterone acetate. IUD=intrauterine device. LNG=levonorgestrel.

* p values calculated using Fisher's exact test except for those for death, which were calculated using the log-rank test.

† Adverse events occurring in >0·5% participants overall or within any single group are listed.

255 pregnancies occurred: 61 (24%) in the DMPA-IM group, 116 (45%) in the copper IUD group, and 78 (31%) in the LNG implant group. 181 (71%) occurred after discontinuation of the randomly assigned method (appendix pp 21, 24). In continuous use analysis, pregnancy incidence was 0·61 per 100 woman-years (95% CI 0·36–0·96) in the DMPA-IM group, 1·11 per 100 woman-years (0·77–1·55) in the copper IUD group, and 0·63 per 100 woman-years (0·39–0·96) in the LNG implant group; the two hormonal methods had lower pregnancy incidence than the copper IUD (p=0·027 for DMPA-IM and p=0·042 for the LNG implant).

## Discussion

This multicountry randomised trial measured HIV incidence among African women assigned to one of three highly effective contraceptive methods. Acceptance of the randomly assigned method, contraceptive continuation, and retention were very high across all methods. HIV incidence was high for all three groups. Fewer women assigned the LNG implant acquired HIV than those assigned to DMPA-IM and the copper IUD, both of which had similar numbers of incident HIV infections. In the primary, modified intention-to-treat analysis, differences in HIV incidence were not substantial or significant across groups.

We designed this trial to detect a 50% increase in HIV incidence for each of the contraceptive methods compared to each of the others. None of the comparisons in the primary modified intention-to-treat analysis showed a 50% increase in HIV incidence, and, under the design of this study an observed approximately 30% increase in HIV incidence would have been found to be statistically significant and HRs less than approximately 1·17 would have upper limits of the 96% CIs that would have ruled out a 50% increase. Results from the primary modified intention-to-treat and continuous use analyses had consistent findings for all comparisons. HIV risk was similar for DMPA-IM and the copper IUD. Both DMPA-IM and the copper IUD had point estimates within a range from 1·18 to 1·29 compared with the LNG implant, in modified intention-to-treat and sensitivity analyses, although the CIs included both the null (ie, no difference) as well as a 50% increase. Viewed from the opposite perspective, the CI for the LNG implant compared with both DMPA-IM and the copper IUD included both the null as well as a 33% decrease. Although this trial had low statistical power to detect an increase in HIV incidence of less than 30%, for individual women at very high HIV risk, we acknowledge that even a relatively small effect might be important in contraceptive and HIV prevention decision making.

In this trial, women randomly assigned to DMPA-IM were less likely to remain on continuous treatment and slightly more likely to be lost to follow-up than those allocated to the other two methods; we also documented post-randomisation differences in self-reported HIV risk-taking behaviours between groups. For this reason, an analysis that censored participants when they ceased continuous use of their randomly assigned contraceptive method but did not use causal methods and adjustment for covariates would be at risk of bias.^20^ When we incorporated inverse probability of censoring weights and time-varying covariate effects to account for likely confounding, the estimated effects were not significant and were also consistent with the modified intention-to-treat analysis results.

Some self-reported sexual behaviours differed among the groups during follow-up, which might reflect biological differences from the contraceptive methods,^21^ behavioural changes due to method-related counselling, or differential reporting. However, absolute differences were small and causal analyses adjusting for sexual behaviours generated similar results to the primary modified intention-to-treat findings. A direct effect of the contraceptive methods on behaviour is plausible because the differences found in our trial were consistent with findings from previous randomised trials in which differential counselling was unlikely, because HIV was not a focus of those trials;^22^ direct biological effects on behaviour would be integral to the method effects whether in a trial setting or not.

All three contraceptive methods were well tolerated, with less than 4% of participants in any group reporting any serious adverse event and less than 9% reporting adverse events resulting in method discontinuation. Adverse events resulting in method discontinuation were generally within the spectrum of common side-effects for these methods. Fewer women using DMPA-IM discontinued their method because of adverse events than women using either the copper IUD or the LNG implant. All three methods had high contraceptive effectiveness (pregnancy rate approximately 1% or less per year) in continuous use analyses. In this study, DMPA-IM users had lower pregnancy rates and higher method continuation than commonly cited typical use estimates.^23^ We recognise that regular counselling, scheduled follow-up, on-site DMPA-IM administration, and clinical management of contraceptive side-effects in this study contributed to high method continuation.

For logistical and financial feasibility, we chose to include three highly effective contraceptive methods available in the African region, including one non-hormonal and two different progestin-only methods. Laboratory studies have shown that different progestins have different biological effects,^24^ and our results might not extend to methods that we did not evaluate. In an effort to maximise the generalisability of the results, we recruited women from community settings from four countries in east and southern Africa, including nine sites across South Africa. Although reported use of DMPA-IM during the 6 months before enrolment was an exclusion criterion, we found that approximately 13% of women had blood medroxyprogesterone acetate concentrations consistent with likely use during this period; similar frequencies of under-reporting of recent contraceptive use have been seen in other studies.^25^, ^26^

We highlight a limitation of this trial that the results only demonstrate the risk of HIV associated with the use of DMPA-IM compared with use of a copper IUD and an LNG implant. Our results cannot be generalised to other contraceptive methods not included in the study, including oral contraceptive pills, alternative injectable methods such as norethisterone enanthate or subcutaneously delivered DMPA, hormone-containing IUDs, or other methods. We enrolled women who desired effective contraception and did not include a placebo or no contraceptive group in this trial. Most of the previous observational studies examining the risk of HIV acquisition compared DMPA-IM users with women not using hormonal contraception and generally not using other forms of highly effective contraception. Although, from a scientific standpoint, it might have been interesting to compare the risk of HIV acquisition associated with use of DMPA-IM (as well as the copper IUD and LNG implant) to no use of contraception, we believe it would have been unethical to do so, as women enrolling in this trial desired effective contraception and such a comparison would not be relevant for such women wishing to use effective contraception. Indeed, for women desiring effective contraception, the salient question is weighing the relative risks and benefits of different methods, not no method.

In many settings in Africa, contraceptive choice is limited, and DMPA-IM is widely used. Copper IUDs are highly effective, non-hormonal, and widely used worldwide, but their use has diminished in Africa; our data provide some of the most robust evidence for clinical safety and contraceptive effectiveness from the region. Inclusion of an LNG implant in this trial was important because implants are highly effective, long acting, and increasingly being used in Africa, and LNG is widely used in oral contraceptive pills and multipurpose prevention technologies in development.^27^, ^28^ This trial demonstrates that delivery of high-quality copper IUD and LNG implant services across multiple African settings is possible with appropriate investment in training, assurance of provider clinical competency, adequate human resources for counselling and management of side-effects, and necessary logistical support including management of commodities.

Despite an individualised HIV prevention package provided to all participants throughout follow-up and country-wide HIV treatment and prevention programmes, HIV incidence was alarmingly high in this population throughout the course of the trial. STI prevalence at baseline was also very high. Women were recruited for this trial on the basis of geography but not other characteristics of HIV risk, such as transactional sex, history of STIs, or self-reported high-risk behaviours. None of the contraceptive methods that we evaluated was designed to be protective against HIV. Our results strongly emphasise the need for more aggressive HIV and STI prevention and management efforts for African women, including PrEP and HIV prevention integrated with contraceptive services.

Women in Africa continue to be at high risk for HIV infection and for morbidity and mortality from unintended pregnancy. 30 years of research has suggested a potential association between hormonal contraceptive use and HIV acquisition risk. This randomised trial did not find a substantial difference in HIV risk among the methods evaluated, and all methods were safe and highly effective. These results underscore the importance of continued and increased access to these three contraceptive methods, as well as expanded contraceptive choices, complemented by high-quality HIV and STI prevention services. Women's informed choice in sexual and reproductive health services is essential. This evidence will enhance women's contraceptive decision making and assist providers and policy makers in delivering high-quality, rights-based contraceptive care.

The ECHO trial is dedicated to the memory of Ward Cates

**This online publication has been corrected. The corrected version first appeared at thelancet.com on June 13, 2019**

## Data sharing

Individual participant data that underlie the results reported in this article, after deidentification, are available for this article. Data will be available beginning 3 months following article publication ending 36 months. The study protocol is available upon publication. The statistical code is available 3 months after publication from the corresponding author. Data is available for researchers who provide a methodologically sound proposal, which will be reviewed by the ECHO Management Committee. Proposals should be directed to icrc@uw.edu; to gain access, data requestors will need to sign a data access agreement and any proposal will require approval by the ECHO Management Committee.
