# Department of Error

**DOI:** 10.1016/S0140-6736(19)31408-4

**Published:** 2019-07-27

*Evidence for Contraceptive Options and HIV Outcomes (ECHO) Trial Consortium. HIV incidence among women using intramuscular depot medroxyprogesterone acetate, a copper intrauterine device, or a levonorgestrel implant for contraception: a randomised, multicentre, open-label trial.* Lancet *2019; http://dx.doi.org/10.1016/S0140-6736(19)31288-7*. On page 8 of this Article (published Online First on June 13), the last sentence of the first paragraph should be “Although this trial had low statistical power to detect an increase in HIV incidence of less than 30%, for individual women at very high HIV risk, we acknowledge that even a relatively small effect might be important in contraceptive and HIV prevention decision making.” This correction has been made to the online version as of June 13, and will be made to the printed Article.

