# Supplementary material for: HIV incidence among women using intramuscular depot medroxyprogesterone acetate, a copper intrauterine device, or a levonorgestrel implant for contraception: a randomised, multicentre, open-label trial
Source: Lancet. 2019 Jul 27;394(10195):303–13. doi: 10.1016/S0140-6736(19)31288-7 (PMC6675739; doi:10.1016/S0140-6736(19)31288-7)
Supplement: Supplementary appendix [file mmc1.pdf]

THE LANCET

Supplementary appendix

This appendix formed part of the original submission and has been peer reviewed. We post it as supplied by the authors.

Supplement to: Evidence for Contraceptive Options and HIV Outcomes (ECHO) Trial Consortium. HIV incidence among women using intramuscular depot medroxyprogesterone acetate, a copper intrauterine device, or a levonorgestrel implant for contraception: a randomised, multicentre, open-label trial. *Lancet* 2019; published online June 13. http://dx.doi.org/10.1016/S0140-6736(19)31288-7.

SUPPLEMENTARY MATERIAL

Funding: The ECHO Trial was made possible by the combined generous support of the Bill & Melinda Gates Foundation, the American people through the United States Agency for International Development and the President's Emergency Plan for AIDS Relief, the Swedish International Development Cooperation Agency as part of the EDCTP2 programme supported by the European Union, the South African Medical Research Council, and the United Nations Population Fund. Contraceptive supplies were donated by the Government of South Africa and United States Agency for International Development. The content is solely the responsibility of the authors and does not necessarily represent the views, decisions, or policies of the institutions with which they are affiliated, the ECHO Trial funders, or the supporting governments.

Trial registration: ClinicalTrials.gov number NCT02550067

Table of Contents, Supplementary Material

Supplementary Material	Page
The ECHO Trial Consortium	3-10
Table S1. Trial inclusion and exclusion criteria	11
Table S2. Operational performance metrics	12
Table S3. Institutional Review Boards/Ethics Committees & Regulatory Review	13
Table S4. Study visits and procedures	14-15
Table S5. Laboratory tests used	16-17
Table S6. Screening and enrolment	18
Table S7. Enrolment by site	19
Table S8. HIV incidence subgroup analyses, subgroups defined by baseline characteristics	20
Table S9. HIV and pregnancy incidence in intention-to-treat analyses, limited to South Africa sites	21
Table S10. Comparison of HIV incidence, during continuous use, using causal models	22
Table S11. Self-reported sexual behaviours during follow-up	23
Table S12. Incident pregnancy during follow-up	24
Figure S1. Estimating recent use of DMPA-IM	25-26
Figure S2. HIV testing algorithm	27
Figure S3. Pregnancy testing algorithm	28
Figure S4. Enrolment, retention, and outcomes	29

The ECHO Trial Consortium:

ECHO Trial Management Committee: Jared M. Baeten (University of Washington, Seattle, USA), James Kiarie (World Health Organization, Geneva, Switzerland), Timothy D. Mastro (FHI 360, Durham, USA), Nelly R. Mugo (Kenya Medical Research Institute, Nairobi, Kenya & University of Washington, Seattle, USA), Helen Rees (Wits Reproductive Health and HIV Institute, Johannesburg, South Africa)

Coordinating Partners:

FHI 360: Pai-Lien Chen, Florence Carayon-Lefebvre d'Hellencourt, Ward Cates, Tonya Colter, Stephanie Combes, Justin Dash, Jennifer Deese, Maria Fawzy, Kelly Feldman, Sophie Gao, Claudia Hansen, Colleen Macko, Nicole Messenger, Charles Morrison, Kavita Nanda, Ginger Pittman, Pairin Seepolmuang, Kathleen Shears, Douglas Taylor, Nicole Walther, Julia Welch, Irina Yacobson

University of Washington: Jennifer Baugh, Benjamin Browning, Deborah Donnell, Jodi Greathouse, Daphne Hamilton, Harald Haugen, Ellie Hawman, Renee Heffron, Kate B. Heller, Rachel Johnson, John Kearney, Lara Kidoguchi, Margaret Lind, Toni Maddox, Deidra Montoya, Susan Morrison, Kelly Moutsos, Dana Panteleeff, Colin Pappajohn, Rena Patel, Torin Schaafsma, Caitlin Scoville, Randy Stalter, Katherine K. Thomas, Valentine Wanga, David Wilson

Wits Reproductive Health and HIV Institute: Deborah Baron, Reneilwe Boikanyo, Susie Cornell, Clare Dott, Ashleigh Jacques, Nomthandazo Mbandazayo, Kerushini Moodley, Sinazo Pato, Melanie Pleaner, Nkunda Vundamina

World Health Organization: Ian Askew, Joanna Cordeiro, Sharon Phillips, Petrus Steyn, Marleen Temmerman

Study Sites:

Eswatini: Manzini (Family Life Association of Eswatini/ICAP at Columbia University): Jessica Justman, Zelda Nhlabatsi (principal investigators); John Manshimba Bambi, Samkelo Dladla, Siyabonga Dlamini, Ntombifuthi Sunshine Dlamini, Temhlanga Dlamini, Mxolisi Dlamini,

Tholakele Dlamini, Tenele Dlamini, Zandile Dlamini, Lunga Dlamini, Dumisani Levy Mabila, Thembinkosi Mabuza, Veli Madau, Zanele Magagula, Tasengudzeni Mamba, John Motsa, Thulani Moyo, Ritha Ncube-Sihlongonyane, Nozipho Ndlovu, Zwide Ndwandwe, Chipo Ngayirongwe, Lainah Njowa-Khumalo, Phindile Nkambule, Nana Nsingo, Harriet Nuwagaba-Biribonwoha, Neena Philip, Ruben Sahabo, Sidumiso Sibanda, Chelsea Solmo, Ntombizonke Tembe, Berthe Yankurije, Thandazile Zikalala, Bhangazi Zwane

Kenya: Kisumu (Kenya Medical Research Institute – Research Care and Training Program): Elizabeth Bukusi, Maricianah Onono (principal investigators); Ken Abuka, Chrispine Abuya, Maqline Achola, Cynthia Akinyi, Linda Akoth, Emily Anyango, Rennah Anyango, Janet Anyango, Joseph Ayoo, Jacktone Hamisi, Lizzie N Kabete, Violet Kwacha, Reina Lenturkana, Joyce Linda, Jackline Majuma, Lyna Memo, Robai Mituyi, Charles Muga, Millicent Muhonja, Simon Mutua, Catherine Mwakio, Imelda Nambale, Lydia Nguti, Petronila Njenga, Stella Njuguna, Elizabeth Nyaranga, Gilbert Nyauke, Sarah Obaje, Charles Ochulo, John Odhiambo, Beryl Ogutu, Nollyne Okuku, Ted Okuku, Florence Ondiek, Patricia Ong'wen, Joan Ongere, Maricianah Onono, Joseph Onyang, Kevin Onyango, Consolata Opondo, Annette Opondo, Davis Ouma, Nelson Ouma, Dismas Ouma, Mildred Owega, Moses Siaji, Nobert Walusala, Sheila Wanyanga, Imeldah Wakhungu

South Africa: Brits (Madibeng Centre for Research): Cheryl Louw (principal investigator); Rose Baaitjies, Christinah Bodigelo, Vuyo Damaneite, Marthie de Villiers, Ben-Matthys Duminy, Emma Hlaletwa, Leonie Jansen van Vuuren, Julia Kekana, Brenda Khoza, Keitumetse Kolobe, Sieglinde Kruger, Penelope Lekala, Winnie Letlhake, Angeline Letsapa, Ilze Louw, Sarah Lusale, Nomhle Mabuza, Ouma Machakela, Letlhogonolo Maebana, Mamphi Mafoka, Tshegofatso Magakwe, Ephenia Maleka, Martha Martins, Selina Mashabane, Jane Mashigo, Rose Masilo, Alinah Masiu, Lenah Masombuka, Blessing Matsunyane, Phumzile Mbatha, Themba Mema, Boitumelo Milanzi, Merisha Minnaar, Gloria Mmoledi, Sarah Moloto, Ester Morodi, Bernard Motau, July Mothabazimbi, Diana Motshweni, Cathy Neate, Phindulo Nemavhidi, Bontle Ntshole,

Christina Olivier, Christinah Phalali, Philemon Ramoshaba, Nilayum Reddy, Maria Sefanyetso, Mamohau Sekonyana, Mavis Senne, Mmule Sethoana, Lorraine Shibambu, Nomonde Sibabela, Zona Sithetho, Tebogo Tlamama, Hanlie Veldsman, Sue Vermaak

South Africa: Cape Town (Emavundleni Research Centre – University of Cape Town, Desmond Tutu HIV Centre): Linda Gail Bekker, Gonasagrie Nair (principal investigators); Shakeera Arnolds, Banzi Bam, Mary Nyasha Chidanyika, Daniella Crida, Nwabisa Danster, Pamela Dukwe, Livhuwani Farisani, Llewelyn Fleurs, Thobeka Gcowa, Samantha Geduld, Jenifer Gelant, Litha Gogo, Sibusiso Gumede, Winnifred Gumula, Xolani Gxako, Kim Hall, Zibelezenkosi Hlebani, Ridley Howard, Amina Ismail, Ebrahiema Jacobs, Lauren Jennings, Ladonna Kapa, Sheetal Kassim, Noluthando Khiya, Ntomboxolo Kwatsha, Justine Lubamba, Melanie Machlachlan, Nocwaka Magobiane, Ferial Mahed, Pamela Makhamba, Macky Matanda, Nombongo Mayekiso, Thembi Mayile, Nozizcelo Mbiza, Zandile Mkhize, Bukiwe Mngqebisa, Noluxolo Mngxekeza, Zanoxolo Mthethi, Julio Muller, Priscila Mvinjelwa, Goodness Zoh Mvuyane, Norah Nandundu, Ndiseka Nashwa, Nontshukumo Ngqabe, Siyabaonga Ngqabe, Sisa Nobanda, Mluleki Nompaondwana, Busiwe Notshokovu, Jennifer Okwundu, Doeriyah Reynolds, Nobubele Rini, Nokwayintombi Scotch, Elaine Sebastian, Ciska Thompson, Thandokazi Tofile, Nomvuselelo Tshongoyi, Abigail Van Der Linde, Dorothie Van Der Vendt, Jabulisile Zuma

South Africa: Durban (MatCH Research Unit [MRU] Commercial City – Department of Obstetrics and Gynaecolgy, Faculty of Health Sciences, University of the Witwatersrand): Mags Beksinska, Jenni Smit (principal investigators); Tatum Arnolds, Ivana Beesham, Smangele Bhengu, Gugu Bhengu, Francisca Ceaser, Sthe Chonco, Nicole Dear, Nqubeko Dladla, Nomusa Dladla, Shannon Evans, Ross Greener, Kavish Harilall, Manjeetha Jaggernath, Sizakele Khanyile, Mbali Khomo, Audrey Khumalo, Ntuthuko Khumalo, Mthokozisi khuzwayo, Amanda Khuzwayo, Brenda Khuzwayo, Muriel Kubheka, Andile Kweza, Londiwe Luthuli, londiwe Mabanga, Khanyisile Mabhida, Thandeka Madiya, Nkosinathi Makhoba, Virginia Maphumulo, Nothando Mhlaba, Cecilia Milford, Nompumelelo Mnikathi, Nonhlanhla Mphili, Nokwanda

Msawoni, Sanelisiwe Msomi, Nomkhosi Mtshali, Skhumbuzo Mzimela, Thiloshnee Naicker, Marlene Naidoo, Tholakele Ndala, Princess Ndwandwe, Keshlin Pillay, Sharon Reddy, Charlott Shangase, Savannah Shezi, Prashant Shoeshanker, Sibusiso Sibiya, Samkelisiwe Sibiya, Keri Singh, Thandeka Sithole, Tshana Watkins, Nokwazi Zungu

South Africa: East London (Effective Care Research Unit – Universities of the Witwatersrand/Fort Hare/Walter Sisulu, Eastern Cape Department of Health): G. Justus Hofmeyr & Mandisa Singata-Madliki (principal investigators); Joanne Batting, Nonzame Cologu, Zwabantu Gqoboka, Bridget Kroger, Thembeka Lekota, Nolukhanya Madliki, Siyabonga Majija, Xoliswa Manyisana, Erica Mashalaba, Portia Mbada, Lulamile Mbongo, Mandisa Mdingi, Pumla Mlotana, Nomthandzo Monakali, Mlungisi Mxesibe, Soyama Ngcozela, Pamela Njikelana, Bulelwa Nogidela-Makhutha, Noxhanti Nondlwana, Catherine Parker, Nontembeko Sibandile, Nombulelo Yutu

South Africa: Edendale (MatCH Research Unit [MRU] Edendale –Department of Obstetrics and Gynaecolgy, Faculty of Health Sciences, University of the Witwatersrand): Jenni Smit (principal investigator); Ivana Beesham, Mags Beksinska, Nqubelo Dladla, Scorns Dlamini, Thuleleni Dlungwana, Sindile Duma, Cynthia Gama, Ross Greener, Thando Gwetu, Tito Hlophe, Manjeetha Jaggernath, Portia Khanyile, Rose Khumalo, Duduzile Lembethe, Zonke Mabude, Nqabisa Madlala, Lungile Maphumulo, Nwabisa Matshoba, Silindile Mbhele, Dumisile Mbhele, Nonduduzo Mfiki, Cecilia Milford, Nompumelelo Mnikathi, Zenhlanhla Mnyandu, Thabisile Molefe, Nolwazi Mthembu, Thiloshnee Naicker, Marlene Naidoo, Princess Ndwandwe, Sindisiwe Nene, Thulisile Ngcobo, Mdu Ngesi, Claudia Ngoloyi, Libuile Ngubo, Keshlin Pillay, Prashant Shoeshanker, Keri Singh, Thandeka Sithole, Khanyisile Sosibo, Nonkululeko Tesfay, Busie Thabathe, Nokuthula Zama, Onesimo Zamla, Portia Zondi, Thobeka Zondi, Celiwe Zondi, Delisile Zondi, Mirriam Zwane

South Africa: Johannesburg (Wits Reproductive Health and HIV Institute (Wits RHI) - University of the Witwatersrand): Thesla Palanee-Phillips (principal investigator); Motsamai

Anthony Alphale, Nombuso Jacqueline Blose, Rutendo Bothma, Robyn Helen Bruce, Fezile Buthelezi, Nazneen Cassim, Busisiwe Cebekhulu, Kgagamatso Chimelwane, Vutomi Cibi, Shameen Cozzi, Clare Dott, Portia Ignatia Makhosazane Duma, Promise Duma, Lizzy Gama, Nerusha Govender, Menzele Shumi Sibusisiwe Thembani Gumede, Ashleigh Catherine Jacques, Emily Kekana, Dineo Khakhane, Themba Langa, Phumelele Nokuthula Langa, Brendley Tebogo Leremi, Mata Ritchie Longwango, Irene Lukas, Nkosana Donel Mabuya, Moshukutjoane Lebogang Maila, Kgabo Phineas Makgoka, Temantfulini Mama Nhassengo, Mosidi Maraba, Romeo Martin, Mainganya Daphney Mashapa, Mmathabo Nnana Mashego, Gwendoline Thotele Refilwe Mashinini, Florence Tintswalo Mathebula, Matheus Mathipa, Eleanor Agnes Matta, Nonkululeko Precious Mayisela, Noncedo Janice Mbatha, Constance Seanokeng Mbatha, Dorica Mbewe, Nomfundo Dahlia Menzi, Elizabeth Gugu Mlangeni, Thembisile Wilmah Mogodiri, Thembisile Wilmah Mogodiri, Maseponki Cecila Mokoena, Semakaleng Irene Mokwena, Willie Monyathabeng, Kerushini Moodley, Sophie Nomsa Morudu, Hlengiwe Theodora Mposula, Emmanule Sinothi Mthalane, Magdeline Judith Mthethwa, Sicelo Samuel Mthimkhulu, Angeline Doreen Nonhlanhla Mzolo, Anika Naidoo, Sandy Naidoo, Ishana Naidoo, Renissa Naidoo, Yuthika Naidoo, Duduzile Ethel Ncube, Nontokozo Happiness Ndlovu, Dikeledi Nhlapo, Lindelwa Nkangane, Ncedo Brian Ntlatleng, Tsitsi Nyamuzihwa, Angel Tinny Nyathi, Levanya Paramanund, Gabisile Nonhlanhla Phala, Mankwana Pheida Ramafalo, Keneoe Maphuti Rampai, Hlalifi Sylvia Rampyapedi, Rebone Frangelina Rebone, Krishnaveni Reddy, Aviva Ruch, Tshepo Jimmy Sagela, Mogobalale Corlett Shogole, Sylvia Sibongile Sibeko, Ayanda Purity Simelane, Vuyane Sizane, Reginah Stuurman, Constance Lebo Thatelo, Mpho Tshabalala, Andile Premrose Twala, Amukelani Califonia Vuma, Victoria Busisiwe Zuma

South Africa: Klerksdorp (The Aurum Institute – Klerksdorp Clinical Research Centre): Raesibe Agnes Pearl Selepe (principal investigator); Nasreen Ahmed, Kagisho Baepanye, Kesenogile Edna Baepanye, Vutomi Cibi, Ken Clarke, Theunis Cloete, Marelize Collignon, Audrey Lebohang Dhlamini, Vinodh A Edward, Candice Eyre, Moogo Fikizolo, Welseh Phindile Galane,

Aaliya Gangat, Thelma Goliath, James Craig Innes, Elizabeth Barbara (Elba) Janse Van Rensburg, Bonita Janse Van Rensburg, Ntebeng Kalauba, Boitumelo Sophy Kekana, Marietjie King, Adriane Kock, Elbie Koetzee, Ndlela Israel Kunene, Aneesa Lakhi, Nondumiso Langa, Hildah Ledwaba, Thelma Liversage, Immaculate Mabasa, Tshegofatso Mabe, Blossom Makhubalo, Heeran Makkan, Mpho Makoanyane, Juanita Market, Richard Nteleki, Selvy Samoele Matshego, Nontsikelelo Mbipha, Zamakhabako Mhlanga, Karabo Moche, Sylvester Modipa, Nthabiseng Mofube, Tshepiso Nkonyana Monakale, Mmaphuti Morifi, Palesa Mosweu, Pontsho Motsatse, Morongoenyane Josephine Motsiri, Dorothy Müller, Wiscar Ndhlovu, Nhlamulo Ndlovu, Maryna Nel, Phindulo Nemavhidi, Lindiwe Nhlangulela, Tanya Nielson, Tedrius Ntshauba, Amukelani Dolly Nyathi, Thandiwe Papalagae, Bertha Pei, Pearl Nomsa Sanyaka, Letlhogonolo Seabela, Melissa Neo Senne, Constance Takavamanya, Maria Mmanare Tlhapi, Amanda Van Aswegen, Constance Van Wyk

South Africa: Ladysmith (Qhakaza Mbokodo Research Clinic): Sydney Sibiya (principal investigator); Rosemary Barnes-Makhanya, Sushell Behariram, Lizanie Downs, Mirriam Dubazane, Brian Groenewald, Jackie Hills, Dudu Hlatshwayo, Thembani Hlayisa, Mandy Hlongwa, Zaheda Ismail, Phila Khambule, Ntombi Khoza, Thabile Khumalo, Phillip Kotze, Nasima Lyons, Nokuphiwa Maketlo, Thulani Mazibuko, Nadia McMaster, Nomonde Mtshali, Isaac Ngema, Patrick Nkosi, Zola Rasimeni, Maryna Schoeman

South Africa: Soshanguve (Setshaba Research Centre): Khatija Ahmed (principal investigator); Veronique Bailey, Morapedi Black, Lydia Chauke, Dineo Chauke, Tlou Chephe, Nkgau Chipape, Keitumetse Diphoko, Lizzie Ditabo, Kreshalen Govender, Makoma Kenoshi, Rebecca Kgatlhe, Joyce Kgole, Millicent Khumalo, Nelia Kubyana, Ziphozonke Langatshe, Muriel Lubethe, Kgomotso Mabidikama, Bongile Mabilane, Martina Madlala, Kedibone Mahlangu, Tercia Makhaphiedza, Simangaliso Makubu, Setty Makunyane, Mookho Malahleha, Tsholofelo Malapane, Vongane Maluleke, Katlego Mapetla, Nomthandazo Maphanga, Josephine Masemola, Rachel Masemola, Mduduzi Masilela, Thandi Masina, Yvonne Mathibedi, Hosea Matlebjane,

Bella Matlonya, Ellen Matsane, Enough Mbatsane, Kagiso Mfomme, Esther Modise, Ntebaleng Mogase, Buskien Mohale, Thamaris Mohapi, Kgaogelo Molapo, Connie Monedi, Edward Moroke, Matshidiso Morolo, Molebogeng Moshiga, Kagiso Mothwa, Thulisile Motshweni, Lebogang Mpete, Margaret Mphahlele, Naledi Ndinisa, Mercy Ndlovu, Martha Ndlovu, Ellen Netshandama, Catherine Ntshane, Ulender Nzimande, Eunice Obonyo, Ayman Osman, Sherman Padayachee, Kgomotso Phohu, Mosidi Pitsoane, Rose Rakolle, Elizabeth Rammutla, Sinah Shaba, Thula Siluma, Joseph Skhosana, Elizabeth Skhosana, Dineo Thindisa, Tsholofelo Thiyo, Zamanxele Tladi, Bonolo Tladinyane, Nadia Van Niekerk

Zambia: Lusaka (UNC Global Projects Zambia – Kamwala Clinic – University of North Carolina at Chapel Hill): Magaret Phiri Kasaro, Jeffrey Stringer (principal investigators); Esther Banda Chabu, Rose C. Chanda, Roma Chilengi, Modesta Zulu Chileshe, Boniface Chileshe, Manze Chinyama, Gabriel Chipili, Megrace Chula, Bethnay Freeman, Elina M. Haafunda, Loveness Kambukwe, Nachengwa Kooma, Calisto Longwe, Chewe C. Lupunga, Nancy Nakweti Machai, Muyaba Joyela Mudenda, Helen B. Mulenga, Fanny Munthali, Dorothy S. L. Muyangwa, Selina Mvula, Humphrey Mwape, Collins K. Mweemba, Monty M. Mweemba, Florence Namukulwa, Ornan Nikora, Agnes Phiri Nkunika, Muyunda M. Pepino, Mary Phiri, Joni Price, Saphira Mwale Sakala, Ntazana Sindano, Sangwani K. Tembo, Bellington Vwalika, Laston Zulu, Dorothy Munkombwe Muchende

Collaborating Partners:

International Centre for Reproductive Health – Kenya & Technical University of Mombasa: Peter Gichangi

University of Zimbabwe: Tsungai Chipato

Collaborating Laboratories: *BARC SA:* Zowie-lea Bagley, Deborah Dart, Lindy Leisegang, Ellisha Maharaj, Samantha Matias, Peter Meewes, Jolanda Truter, Raqual Viana, Carole Wallis; *Partners in Health Research & Development – Clinical Trials Research Laboratory:* Bhavna Chohan, Mary Kibatha, Marion Kiguoya-Njau, James Munyao, Euticus Mwangi, Carol Senoga;

Oregon Health Sciences University – Endocrine Technologies Core – Oregon National Primate Research Center: David W. Erikson, Steven W. Blue, Amy V. Kaucher

Clinical Laboratory Services, University of the Witwatersrand (laboratory oversight): Elongo Fritz, Shamim Hoosen, Ute Jentsch, Nomfundo Maduna, Evasen Raju, Shiksha Reddy

DF/Net Research, Inc. (data management): Lisa Ondrejcek, Darryl Pahl, Khristina Kline, Eric Bosch, Zoe Bowen, Lydia Browne, Ann Bryan, Barry Curtis, Lori Filipcic, Donna Fulcher, Julie Gilmour, Alexandra Halsey, Kevin Heins, Brianna Heins, Pauline Houx, Trevor Hyde, Matt Johnson, Sandra Jurilj-Cankusic, Neeta Kabra, Jia Jin Kee, Phil Kirsch, Bill Larson, George LaTorre, Tadas Lukosevicius, Yonghong Luo, Clare Mengebier, Corey Miller, Joseph Murphy, Camille Pahl, Eleanor Pahl, Jean Paul Pease, Krysta Pendleton, Mark Ponti, Jerad Post, Brian Postle, Bazel Rivera, Sara Seo, Lauren Solmssen, Kristen Sukraw, Rheanna Summers, Taura Vastakas, Mark Wessel, Angela Williams, Edith Wolff

Randomize.net (study method randomization): Nick Willan

Clinical Monitors: Waheeda Bux, Savi Chetty-Tulsee, Ahmed Goolam, Tharnija Lalbahadur, Hilda O'Hara

Contraceptive consultants: Modiege (Didi) Mojapelo, Margaret Moss

Independent

Community Advisory Boards at each trial site

Global Community Advisory Group: Juliet Akumu, Emily Bass, Georgina Caswell, Tyler Crone, Anna Forbes, Margaret Happy, Sibongile Maseko, Engwase Mwale, Lilian Mworeko, Definate Nhamo, Njoki Otieno, Yvette Raphael, Siphiwe Vilakati, Jacqui Wambui

HIV Endpoints Committee: Connie Celum, Robert Coombs, François Venter

Data and Safety Monitoring Board: Thomas Fleming (chair), Anne Buvé, Anne-Beatrice Kihara, Dorothy Mbori-Ngacha, Tarek Meguid, Herbert Peterson, Sengeziwe Sibeko

Table S1. Trial inclusion and exclusion criteria

**Recruitment**	Women were recruited from family planning/reproductive health clinics, clinics serving post-partum and post-abortion clients, other relevant clinics, and the general community.
**Inclusion Criteria**	To be eligible for the study a woman must have met all of the following criteria:<br>• 16-35 years of age<br>o Previously pregnant 16 and 17 year olds, where permissible by national regulations and local IRB approval<br>• HIV-seronegative<br>• Wants to use effective contraception<br>• Is able and willing to provide written informed consent<br>• Agrees to be randomised to either DMPA, LNG implant, or copper IUD<br>• Agrees to use assigned method for 18 months<br>• Agrees to follow all study requirements<br>• Intends to stay in the study area for the next 18 months, and willing and able to provide adequate locator information<br>• If has had a recent third trimester birth, is at least 6 weeks postpartum at time of enrolment<br>• Is sexually active (has had vaginal sex within the last 3 months) or was pregnant within the last 3 months<br>• Agrees not to participate in studies of drugs or vaccines or any other clinical research study while participating in this study.
**Exclusion Criteria**	A woman who reported or was found to have any of the following criteria was excluded from the study:<br>• Medical contraindications (Category 3 or 4 criteria as detailed in the World Health Organization *Medical eligibility criteria for contraceptive use (*5th ed 2015, Geneva: WHO) to DMPA, LNG implant, or copper IUDs, including<br>o Persistent measured blood pressure 160/100 mmHg or higher, or history of uncontrolled hypertension due to vascular disease<br>o History of a stroke or heart attack<br>o History of rheumatic disease such as lupus, with positive or unknown antiphospholipid antibodies or low platelet count<br>o Multiple risk factors for cardiovascular disease including two or more of: a) smoked regularly for the past 6 months, b) history of hypertension, c) history of diabetes, and d) history of abnormal cholesterol/lipid disorder<br>o Diabetes for more than 20 years or diabetes with nephropathy, retinopathy, or neuropathy<br>o A current/acute blood clot in her legs or lungs<br>o History of severe liver cirrhosis or a liver tumor<br>o Current or previous breast cancer<br>o Endometrial, ovarian, or cervical cancer<br>o History of trophoblastic disease<br>o Unexplained vaginal bleeding between menstrual periods or bleeding after intercourse<br>o History of pelvic tuberculosis<br>o Known anatomical abnormality of the uterus incompatible with IUD insertion<br>o A recent septic abortion<br>o Untreated mucopurulent cervicitis on exam, untreated pelvic inflammatory disease (PID), or untreated known gonorrhoea or chlamydia<br>• Has received a DMPA or NET-En injection in the last 6 months<br>• Has used an implant or an IUD in the last 6 months<br>• Is pregnant or intending to become pregnant in the next 18 months<br>• Has had a hysterectomy or sterilization<br>• Has previously enrolled in the study<br>• Has any condition (social or medical), which in the opinion of the investigator, would make study participation unsafe or complicate data interpretation.

Table S2. Operational performance metrics

ECHO Performance Standard	Target	Final Data from the Trial (Overall)
**#1 Accrual**	Achieve target sample within ~18 months	20.5 months
**#2 Method refusal**	<5% of subjects*	0.6%
**#3 Retention**	Per-visit completion of ≥90% and ≤10% of expected woman-years lost*	Per-visit completion 93.6%across all follow-up, 5.3% of expected woman-years lost
**#4 Method discontinuation**	≤10% of all person-time off assigned method*	8.1%
**#5 HIV incidence**	sufficient to meet the study objectives (≥3.5 per 100 woman-years)	3.81 per 100 woman-years
**#6 Ineligible enrollments**	<1-2% of total	0.2%
**#7 HIV endpoint adjudication**	up-to-date for each DSMB review	Met for each review
**#8 Data quality**	current for each DSMB, QC ≤5/100 case report forms (CRFs), fax time ≤7days for ≥95% of CRFs	Met for each review, 3.6 QCs/100 CRFs, 95.5% faxed in ≤7days

* Overall, at each trial site, and within each randomised arm

Table S3. Institutional Review Boards/Ethics Committees & Regulatory Review

Country: Site	Institutional Review Board / Ethics Committee Review<br>*Regulatory Review*
Overall review	FHI 360 Protection of Human Subjects Committee
Eswatini: Manzini	Swaziland Scientific and Ethics Committee for Ministry of Health and Social Welfare Research<br>Columbia University Institutional Review Board
Kenya: Kisumu	Kenya Medical Research Institute Scientific and Ethics Review Unit<br>*Pharmacy and Poisons Board*
South Africa: Brits	University of Witwatersrand Human Research Ethics Committee<br>*South Africa Medicines Control Council (notification)*
South Africa: Cape Town	University of Cape Town Human Research Ethics Committee<br>*South Africa Medicines Control Council (notification)*
South Africa: Durban	University of Witwatersrand Human Research Ethics Committee<br>*South Africa Medicines Control Council (notification)*
South Africa: East London	University of Witwatersrand Human Research Ethics Committee<br>WHO Research Ethics Review Committee<br>*South Africa Medicines Control Council (notification)*
South Africa: Edendale	University of Witwatersrand Human Research Ethics Committee<br>*South Africa Medicines Control Council (notification)*
South Africa: Johannesburg	University of Witwatersrand Human Research Ethics Committee<br>WHO Research Ethics Review Committee<br>*South Africa Medicines Control Council (notification)*
South Africa: Klerksdorp	University of Witwatersrand Human Research Ethics Committee<br>*South Africa Medicines Control Council (notification)*
South Africa: Ladysmith	University of Witwatersrand Human Research Ethics Committee<br>*South Africa Medicines Control Council (notification)*
South Africa: Soshanguve	University of Witwatersrand Human Research Ethics Committee<br>*South Africa Medicines Control Council (notification)*
Zambia: Lusaka	University of Zambia Biomedical Research Ethics Committee<br>*National Health Research Authority at Ministry of Health*<br>*Zambia Medicine Regulatory Authority*

Table S4. Study visits and procedures

	SCR	ENR	MTH 1	QTRYL Y	FINAL	POSSIBLE SEROCONVERSION
**ADMINISTRATIVE AND REGULATORY**						
Written informed consent (include future/addt'l testing)	X	X[1]				
Assignment of participant identification (PTID) numbers	X					
Assessment/ confirmation of eligibility	X	X				
Collection/review/update of locator information	X	X	X	X	X	X
Participant reimbursement	X	X	X	X	X	X
Visit scheduling	X	X	X	X		X
**CONTRACEPTIVE**						
Contraceptive counselling, including continuation counselling	X	X	X	X	X	X
Randomisation and first injection, implant/IUD insertion		X				
Contraceptive side effects assessment			X	X	X	X
Injectable contraceptive provision		X		X	X	X
**BEHAVIOURAL**						
Risk reduction counselling	X	X		X	X	X
HIV pre- and post-test counselling	X			X	X	X
Behavioural data collection		X		X	X	X
**CLINICAL**						
Demographic history	X					
Contraceptive, reproductive history/update	X	X		X	X	X
Physical examination (height, weight, BP)	X			*	*	X
Pregnancy assessment	X			X		
Pelvic examination (including bimanual and/or speculum as indicated and defined in the MOP)	X	IUD only		*	X	X
IUD string check (confirmation by ultrasound if strings not found)			X		X	X
Screen for cervical cancer (optional –based on local standards of care and availability of treatment)	X					
Syndromic assessment for STIs	X	*		*	X	X
AE assessment			X	X	X	X
**LABORATORY**						
Urine pregnancy test	*	X^		*	X^	X^
HIV rapid test	X			X	X	X
HIV confirmatory testing (HIV Western blot and/or HIV EIA, with HIV RNA PCR)						X
HIV RNA and CD4 count[+]				X	X	X
Endocervical swab for STI testing (NAAT for GC, CT)	X				X	X
Endocervical swab for archiving	X				X	X
Cervicovaginal specimen for semen exposure testing					X	X

	SCR	ENR	MTH 1	QTRYL Y	FINAL	POSSIBLE SEROCONVERSION
Plasma and Serum archive (for HIV and HSV-2 testing) [#]		X		X (6M only)	X	X

[1] Only for sites which opt to use separate screening and enrolment consent forms
*as clinically indicated;
^if a woman has had a clinician verified miscarriage or pregnancy termination within 12 days, urine pregnancy test is not needed
[#] HSV-2 testing will be done on enrolment specimens; testing at other visits dependent on additional funding
[+] HIV RNA and CD4 count will be done for confirmed seroconverters

Notes:

- Any visit (screening, enrolment, follow-up, final, seroconversion) can be followed by an as-needed visit to provide laboratory test results (e.g., STI, confirmatory HIV, etc.) that return after that visit and any related counselling and treatment.
- Confirmed HIV-positive women will continue to be followed on the study schedule, with all procedures except on-going HIV serologic testing. HIV RNA and CD4 counts will be done for seroconverters at the time of seroconversion, at subsequent quarterly visits, and at the final study visit. In addition, the enrolment plasma archive sample will be tested by HIV RNA PCR for all HIV seroconverters to define whether seroconverters were infected at the time of enrolment, as described in Section 8.2.1
- SCR = screening; ENR = enrolment; FINAL VISIT = last on-study visit, to permit completion of procedures in the event of early participant or study termination; POSSIBLE SEROCONVERSION = potential HIV seroconversion (i.e., visit at which rapid testing is positive, while confirmatory testing pending)

Table S5. Laboratory tests used

Country: Site	HIV rapid	Pregnancy	*C. trachomatis & N. gonorrhoeae*	HSV-2	HIV EIA	HIV Western blot	HIV RNA	HIV DNA
Eswatini: Manzini	Determine<br>UniGold HIV	QuickVue	GeneXpert<br>RealTime	FOCUS	ARCHITECT	BIO-RAD	RealTime	TaqMan
Kenya: Kisumu	Determine<br>UniGold HIV<br>First Response	QuickVue	Panther	FOCUS	Murex	BIO-RAD	TaqMan<br>RealTime	TaqMan
South Africa: Brits	Determine<br>UniGold HIV	QuickVue	GeneXpert<br>RealTime	FOCUS	ARCHITECT	BIO-RAD	RealTime	TaqMan
South Africa: Cape Town	Determine<br>OraQuick	QuickVue	GeneXpert<br>RealTime	FOCUS	ARCHITECT	BIO-RAD	RealTime	TaqMan
South Africa: Durban	ABON<br>Advanced Quality<br>First Response	QuickVue	GeneXpert<br>RealTime	FOCUS	ARCHITECT	BIO-RAD	RealTime	TaqMan
South Africa: East London	ABON<br>Advanced Quality<br>BioTracer	Atlas	GeneXpert<br>RealTime	FOCUS	ARCHITECT	BIO-RAD	RealTime	TaqMan
South Africa: Edendale	ABON<br>Advanced Quality<br>First Response	QuickVue	GeneXpert<br>RealTime	FOCUS	ARCHITECT	BIO-RAD	RealTime	TaqMan
South Africa: Johannesburg	Determine<br>Advanced Quality	CliniHealth<br>QuickVue	GeneXpert<br>RealTime	FOCUS	ARCHITECT	BIO-RAD	RealTime	TaqMan
South Africa: Klerksdorp	Determine<br>UniGold HIV	CLINITEST<br>QuickVue	GeneXpert<br>RealTime	FOCUS	ARCHITECT	BIO-RAD	RealTime	TaqMan
South Africa: Ladysmith	Determine<br>Advanced Quality	QuickVue<br>Always Accurate	GeneXpert<br>RealTime	FOCUS	ARCHITECT	BIO-RAD	RealTime	TaqMan
South Africa: Soshanguve	Determine<br>UniGold HIV	QuickVue	GeneXpert<br>RealTime	FOCUS	ARCHITECT	BIO-RAD	RealTime	TaqMan
Zambia: Lusaka	Determine<br>SD BIOLINE	QuickVue	LightCycler<br>COBAS<br>RealTime<br>GeneXpert	FOCUS	Elecsys	Consort	AmpliPrep<br>TaqMan	TaqMan<br>AmpliPrep

ABON = ABON HIV 1/2/O Tri-Line
Advanced Quality = Advanced Quality ONE STEP Anti-HIV (1&2)
AmpliPrep = COBAS AmpliPrep
ARCHITECT = Abbott ARCHITECT
Atlas = Atlas hCG One Step
BIO-RAD = BIO-RAD GS HIV-1 Western Blot
BioTracer = BioTracer HIV ½
COBAS = COBAS z480z
Consort = Consort E455 with UV transilluminator
Determine = Alere Determine HIV-1/2
Elecsys = Roche Elecsys HIV combi PT
First Response = First Response HIV 1-2-O
FOCUS = FOCUS HerpeSelect 2 ELISA IgG
GeneXpert = Cepheid GeneXpert
LightCycler = LightCycler 480 II
Murex = Abbott Murex
OraQuick = OraQuick Advance HIV-1/2
Panther = Hologic Panther platform
RealTime = Abbott RealTime
QuickVue = QuickVue OneStep
SD BIOLINE = SD BIOLINE HIV-1/2
TaqMan = Roche COBAS TaqMan

Table S6. Screening and enrolment

Participants Screened	12,750*
Participants Enrolled*	7830 (61.4%)
Participants not Enrolled	4920 (38.6%)
Participant did not complete all screening procedures	409 (8.3%)
Participant is eligible but declined enrollment	1094 (22.2%)
Participant not eligible	3416 (69.4%)
Participant is below age of consent/assent or >35 years	7 (0.2%)
Participant is HIV-infected	1540 (45.1%)
Participant does not want to use study contraceptive methods	99 (2.9%)
Participant is unable / unwilling to provide written informed consent or assent	2 (0.1%)
Participant is <18 years old, and guardian is unable / unwilling to provide written informed consent	1 (0.0%)
Participant declines to be randomized to a study contraceptive	170 (5.0%)
Participant is not sure if she wants to use assigned method for at least 18 months	21 (0.6%)
Participant declines to follow other study requirements	174 (5.1%)
Participant plans for relocation/travel	56 (1.6%)
Participant had third trimester birth <6 weeks ago	9 (0.3%)
Participant has not had vaginal sex nor was pregnant in the last 3 months	142 (4.2%)
Participant currently participating in other clinical research study	5 (0.1%)
Participant has one or more medical contraindications	218 (6.4%)
Participant has used DMPA-IM (or other injectable contraceptive), an implant, or an IUD in the last 6 months	241 (7.1%)
Participant is pregnant	274 (8.0%)
Participant is intending to become pregnant	19 (0.6%)
Participant has had a hysterectomy or sterilization	9 (0.3%)
Other reason, including investigator decision	627 (18.4%)

Table S7. Enrolment by site

		Randomised Group						
**Country**	**Site**	**DMPA-IM**	**%**	**Copper IUD**	**%**	**LNG implant**	**%**	**Total**
Eswatini	Manzini	167	33.3	167	33.3	168	33.5	502
Kenya	Kisumu	299	33.2	301	33.4	301	33.4	901
South Africa	Brits	136	33.4	134	32.9	137	33.7	407
South Africa	Cape Town	187	33.4	186	33.2	187	33.4	560
South Africa	Durban	287	33.3	286	33.2	288	33.4	861
South Africa	East London	205	33.3	205	33.3	205	33.3	615
South Africa	Edendale	205	33.6	204	33.4	202	33.1	611
South Africa	Johannesburg	230	33.0	235	33.7	232	33.3	697
South Africa	Klerksdorp	187	33.7	183	33.0	185	33.3	555
South Africa	Ladysmith	219	33.5	217	33.2	217	33.2	653
South Africa	Soshunguve	269	33.2	269	33.2	272	33.6	810
Zambia	Lusaka	218	33.1	220	33.4	220	33.4	658
**Total**		2609	33.3	2607	33.3	2614	33.4	7830

Table S8: HIV incidence by randomised group, including in subgroups defined by baseline characteristics

For the primary intention-to-treat analysis (Table 2), an analysis of Schoenfeld residuals identified a lack of proportional hazards for comparisons to the copper IUD (p=0·046 for DMPA-IM *vs* copper IUD; p=0·001 for LNG implant *vs* copper IUD). This result was highly affected by a disproportionately low rate of seroconversion in the copper IUD group during the first 3 months of follow-up, and to a lesser extent a disproportionally low rate of infection among LNG implant users returning late for their final (month 18) visit. There was no meaningful evidence for lack of proportional hazards between months 3 and 18 (p>0.1 for all tests of interaction with time), the period over which most infections in all groups occurred (112 [78%] of 143 in the DMPA-IM group, 122 [88%] of 138 in the copper IUD group, and 95 [82%] of 116 in the LNG implant group). Despite the deviation from the proportionality assumption overall, the HR from a Cox model remains a valid estimate of the average comparative risk over study follow-up (similar to a crude incidence ratio).

In the primary intention-to-treat analysis, overall HIV incidence was 3·81 per 100 woman-years (95% CI 3·45–4·21), 4·26 per 100 woman-years (3·77–4·79) in women younger than 25 years, 2·96 per 100 woman-years (2·10–4·07) in those 25 years or older, 3·21 per 100 woman-years (2·74–3·73) in HSV-2-negative women, and 4·54 per 100 woman-years (3·90–5·25) in HSV-2-positive women. In analyses adjusting for site and randomly assigned group, age younger than 25 years was associated with higher HIV incidence than age 25 years or older (HR 1·32 [95% CI 1·05–1·64], p=0·014) and enrolment HSV-2-positive status was associated with higher HIV incidence than HSV-2-negative status (1·14 [1·00–1·30], p=0·057).

	DMPA-IM			Copper IUD			LNG Implant			DMPA-IM v Copper IUD		DMPA-IM v LNG Implant		Copper IUD vs LNG Implant	
	**N**	**N events**	**Rate**	**N**	**N events**	**Rate**	**N**	**N events**	**Rate**	**HR (95% CI)**	**p-value**	**HR (95% CI)**	**p-value**	**HR (95% CI)**	**p-value**
**Gravidity**											0.33		0.93		0.41
Gravid	2,063	111	4.03	2,097	102	3.57	2,129	91	3.16	1.11 (0.85, 1.46)		1.24 (0.94, 1.64)		1.11 (0.84, 1.48)	
Nulligravid	493	32	4.88	474	36	5.64	459	25	4.01	0.85 (0.53, 1.37)		1.21 (0.71, 2.04)		1.42 (0.85, 2.37)	
**BMI**											0.08		0.60		0.25
≤30	1,916	105	4.12	1,912	112	4.30	1,891	86	3.37	0.93 (0.71, 1.21)		1.18 (0.89, 1.57)		1.27 (0.96, 1.68)	
>30	634	38	4.47	658	26	2.90	691	30	3.19	1.53 (0.93, 2.52)		1.37 (0.85, 2.21)		0.89 (0.53, 1.51)	
**Living children**											0.49		0.73		0.32
None	578	39	5.07	556	39	5.20	548	27	3.61	0.91 (0.58, 1.43)		1.33 (0.81, 2.18)		1.46 (0.89, 2.39)	
At least one	1,978	104	3.94	2,015	99	3.60	2,040	89	3.23	1.10 (0.83, 1.44)		1.20 (0.91, 1.60)		1.10 (0.82, 1.46)	
**Living with partner**											0.92		0.31		0.36
No	1,806	126	5.26	1,804	121	4.93	1,832	97	3.93	1.05 (0.82, 1.35)		1.30 (0.99, 1.69)		1.24 (0.95, 1.62)	
Yes	750	17	1.68	767	17	1.62	756	19	1.84	1.01 (0.52, 1.98)		0.90 (0.47, 1.74)		0.89 (0.46, 1.72)	
**Unprotected sex***											0.08		0.34		0.50
Never	699	33	3.51	694	42	4.48	702	31	3.29	0.74 (0.47, 1.17)		1.00 (0.61, 1.64)		1.35 (0.85, 2.16)	
Any	1,856	110	4.46	1,877	96	3.75	1,886	85	3.32	1.18 (0.90, 1.55)		1.32 (1.00, 1.76)		1.12 (0.84, 1.50)	
**New or multiple partners**											0.24		0.90		0.21
No	2,360	122	3.87	2,349	121	3.78	2,402	98	3.01	1.00 (0.77, 1.28)		1.24 (0.95, 1.62)		1.25 (0.96, 1.63)	
Yes	193	21	8.32	220	17	5.77	185	18	7.31	1.50 (0.79, 2.86)		1.19 (0.63, 2.23)		0.79 (0.41, 1.53)	
**Coital acts (3 months prior to enrolment)**											0.47		0.98		0.47
0-9 acts	1,361	87	4.79	1,330	87	4.84	1,368	69	3.75	0.97 (0.72, 1.31)		1.23 (0.90, 1.69)		1.27 (0.93, 1.74)	
9+ acts	1,194	56	3.51	1,241	51	3.00	1,220	47	2.84	1.16 (0.79, 1.70)		1.22 (0.83, 1.81)		1.05 (0.71, 1.57)	
C. trachomatis* or *N. gonorrhoeae											0.73		0.28		0.45
Both negative	2,030	104	3.83	2,022	97	3.51	2,046	76	2.74	1.08 (0.81, 1.42)		1.36 (1.01, 1.84)		1.27 (0.94, 1.71)	
Either/both positive	519	39	5.72	548	41	5.56	537	39	5.40	0.98 (0.63, 1.52)		1.01 (0.65, 1.58)		1.03 (0.67, 1.60)	

HR = hazard ratio, CI = confidence interval

*Never unprotected sex includes participants with no partner, no sex, or having sex but always using a condom in the previous three months. Any unprotected sex includes participants who had sex at least once and never, rarely, sometimes, or often used a condom in the previous three months.

P-values are for whether HR differs by subgroup. HRs, CIs and p-values were generated by adding the subgroup variable to the primary analysis proportional hazards regression model and including an interaction term between randomised group and the subgroup variable; p-values are generated by performing a Likelihood Ratio test on the interaction term and are interpreted to indicate whether there is effect modification by subgroup. Nominal 95% confidence intervals were used for HR estimates across all subgroups, as specified in the trial's Statistical Analysis Plan.

Table S9. HIV and pregnancy incidence in intention-to-treat analyses, limited to South Africa sites

	DMPA-IM			Copper IUD			LNG Implant			DMPA-IM vs Copper IUD		DMPA-IM vs LNG Implant		Copper IUD vs LNG Implant	
	N	N events	Rate*	N	N events	Rate*	N	N events	Rate*	HR (95% CI)	p-value	HR (95% CI)	p-value	HR (95% CI)	p-value
**HIV**	1879	124	4.94	1889	118	4.58	1902	103	4.02	1.05 (0.82, 1.36)	0.69	1.19 (0.91, 1.55)	0.20	1.13 (0.87, 1.47)	0.37
**Pregnancy**	1916	38	1.47	1908	74	2.82	1920	53	2.02	0.51 (0.34, 0.77)	0.0011	0.70 (0.46, 1.08)	0.11	1.37 (0.95, 1.96)	0.089

HR = hazard ratio, CI = confidence interval

Rates are per 100 woman-years.

For this post hoc subset, analyses were done as per Table S8 (for HIV) and Table S12 (for pregnancy).

Table S10. Comparison of HIV incidence, during continuous use, using causal models

Supportive analyses were performed to estimate the causal effects of continuous method use (as defined in the Methods) on risk of HIV among women who initiated their randomized method. To account for potential biases induced by dependent censoring (i.e., stopping use of randomized method or loss to follow-up), stabilized inverse probability of censoring weights (IPCWs) were incorporated into a Cox proportional hazards regression model, stratified by site. The IPCWs were constructed for each quarterly visit where censoring occurred using pooled logistic regression and time-varying covariate data collected at scheduled quarterly visits. When applicable, unscheduled visit data was assigned to the nearest scheduled visit. Time-varying covariates considered when constructing IPCWs included having a primary partner in the previous 3 months, vaginal sex without a condom in the previous 3 months, more than one sex partner, a new sex partner in the previous 3 months, more than one sex partner or a new sex partner in the previous 3 months, vaginal sex in the last 7 days, condom used for last vaginal sex act, and sex for money in the previous 3 months. Of these, only having a primary partner, vaginal sex without a condom and having vaginal sex in the last 7 days were significantly associated with censoring and included in the final weights.Two proportional hazards regression models were fitted incorporating the IPCWs: one that included randomised group and pre-specified baseline covariates (age ≤24, having living children, living with husband or primary partner, vaginal sex without a condom, more than one sex partner, and new sex partner) and one including randomised group, baseline covariates and pre-specified time-varying effects of vaginal sex without a condom, more than one sex partner, and new sex partner.

Our primary intention-to-treat analysis did not identify statistically significant differences in risk of HIV among the randomised groups. In the table, an analysis which does not incorporate weights or time-varying covariate information is reported for comparison purposes; in this analysis, which did not use causal methods to consider biases from dependent censoring, HIV acquisition risk was higher for DMPA-IM compared to the LNG implant, a result that was statistically significant. Although commonly reported in clinical trials, such per-protocol analyses do not result in valid estimates of treatment effects if they fail to account for time-varying factors associated with remaining on treatment and in follow-up and the outcome. Using causal methods and adjusting for baseline and time-varying covariates, all hazard ratios for HIV acquisition comparing among the three randomised methods were not statistically significant and findings were consistent with the intention-to-treat results.

	**DMPA-IM vs Copper IUD**		**DMPA-IM vs LNG Implant**		**Copper IUD vs LNG Implant**	
**Analysis**	**HR (96% CI)**	**p-value**	**HR (96% CI)**	**p-value**	**HR (96% CI)**	**p-value**
Unweighted, adjusted for baseline covariates	1.17 (0.89, 1.53)	0.25	1.34 (1.02, 1.77)	0.029	1.15 (0.88, 1.51)	0.29
Weighted, adjusted for baseline covariates	1.07 (0.82, 1.40)	0.62	1.27 (0.96, 1.68)	0.080	1.19 (0.91, 1.56)	0.19
Weighted, adjusted for baseline and time-varying covariates	1.10 (0.84, 1.44)	0.49	1.29 (0.97, 1.71)	0.060	1.18 (0.90, 1.55)	0.22

Continuous use estimates included only time on study with continuous use of randomized methods, as defined in the Methods, and included data from 7317 women. Causal analysis methods were used to estimate HRs during continuous use, with inverse probability weighting for discontinuation of randomized method, adjusted for baseline covariates (age ≤24, having living children, living with husband or primary partner, vaginal sex without a condom, more than one sex partner, and new sex partner) and time-varying covariates (vaginal sex without a condom, more than one sex partner and new sex partner in the previous 3 months).. Inverse probability weights for discontinuation of randomized method relied on vaginal sex without a condom, vaginal sex last 7 days, and having primary partner in the previous 3 months. Covariate data for calculating weights were missing for 82 (1.1%) of women; and covariates for model adjustment were missing for another 9 (0.1%) of women, corresponding to 0.1% of visits. Women missing censoring weights or covariate data were excluded from the causal analysis.

Table S11. Self-reported sexual behaviours during follow-up

	Randomised Group			p-values		
**Risk behaviour in the prior 3 months**	**DMPA-IM** (12,866 visits)	**Copper IUD** (13,444 visits)	**LNG Implant** (13,541 visits)	**DMPA-IM vs Copper IUD**	**DMPA-IM vs LNG Implant**	**Copper IUD vs LNG Implant**
Multiple sex partners	460 (3.6%)	848 (6.3%)	660 (4.9%)	<0.0001	0.00012	0.0037
New sex partner	381 (3.0%)	717 (5.3%)	537 (4.0%)	<0.0001	0.00038	0.00017
More than 10 vaginal sex acts	6194 (48.2%)	6773 (50.4%)	6616 (48.9%)	0.022	0.29	0.22
Any unprotected sex	8539 (66.4%)	9537 (70.9%)	9391 (69.4%)	<0.0001	0.0014	0.084
No condom used for last sex act	6367 (53.3%)	7146 (56.2%)	7175 (56.5%)	0.0089	0.0023	0.64
Sex for money of gifts	73 (0.6%)	119 (0.9%)	86 (0.6%)	0.027	0.54	0.097
Sex during vaginal bleeding	931 (7.2%)	1226 (9.1%)	965 (7.1%)	<0.0001	0.68	<0.0001

P-values for differences in behavior between randomised groups across all visits were computed using generalised estimating equations with follow-up behaviour as the outcome and randomised group, enrollment site, and baseline behaviour as predictors. Models used the binomial distribution and logit link function. Robust standard errors were used. Accounting for multiple comparisons among behaviors (7 variables, 3 pairs) the Bonferroni corrected p-value for statistical significance is 0.05/21 = 0.0024.

Table S12. Incident pregnancy during follow-up

	DMPA-IM			Copper IUD			LNG Implant			DMPA-IM vs Copper IUD		DMPA-IM vs LNG Implant		Copper IUD vs LNG Implant	
	**N**	**N events**	**Rate***	**N**	**N events**	**Rate***	**N**	**N events**	**Rate***	**HR (95% CI)**	**p-value**	**HR (95% CI)**	**p-value**	**HR (95% CI)**	**p-value**
**Intention-to-treat analysis**	2600	61	1.75	2596	116	3.27	2608	78	2.19	0.52 (0.38, 0.72)	<0.0001	0.78 (0.55, 1.09)	0.15	1.49 (1.11, 1.99)	0.0077
**Continuous use analysis**	2583	18	0.61	2506	35	1.11	2584	21	0.63	0.53 (0.30, 0.93)	0.027	0.93 (0.49, 1.74)	0.81	1.76 (1.02, 3.02)	0.042

HR = hazard ratio, CI = confidence interval

Rates are per 100 woman-years.

Estimated date of fertilization was computed for each pregnancy using a pre-defined standard algorithm (Figure S3). Women determined to be pregnant at enrolment were excluded from both pregnancy analyses; women who acquired HIV were included.

Counting process Cox proportional hazards model was used to account for multiple pregnancies on study. Women are at risk for pregnancy when they were not pregnant, for women with multiple pregnancies time at risk restarted at the date of pregnancy outcome. Continuous use analysis for pregnancy includes only first pregnancies and time to first discontinuation. Causal methods are not used, as pregnancy causes censoring (discontinuation) of randomized contraceptive method.

In the intention-to-treat analysis, all pregnancies occurring after enrolment (including multiple pregnancies for a single woman) were included, and intervals of woman-years at risk of pregnancy excluded time estimated to be pregnant. Hazard ratios with 95% confidence intervals were estimated using a single counting process Cox proportional hazards regression model with three-way class variable for randomisation group.

For the continuous use analysis, woman-years at risk of pregnancy were limited to time women were thought to be using their randomized contraceptive method (computed as time from enrolment to the earlier of first method discontinuation, first pregnancy, or last clinic visit). Hazard ratios were estimated using a single Cox proportional hazards regression model with a three-way class variable for randomisation group.

Figure S1. Estimating recent use of DMPA-IM

There are limited pharmacokinetic (PK) data for DMPA-IM based on dense sampling in the recent literature. What is available is a mix of different formulations, assays, injection sites, sampling times, and populations, any of which may have an impact on estimated PK. However, insightful data on the commercially-available 150 mg DMPA-IM (Pfizer) product exist, including:

- A bioequivalence (BE) study comparing the Pfizer DMPA-IM product to a generic (Gensia Sicor Pharmaceuticals), submitted as part of the NDA for the latter in 2002 [1]. That study, conducted among 122 post-menopausal Caucasian women, reported 3-month MPA troughs of 0.58 ng/mL in the Pfizer group and 0.56 ng/mL in the generic group, and an apparent half-life of approximately 40 days. Coefficients of variation around 3 months were generally between 40% to 50%, and mean MPA concentrations remained above 0.3 ng/mL four months after injection.
- The Pfizer prescribing information for DMPA-IM [2], reporting a half-life of approximately 50 days.
- A randomized efficacy study sponsored by Pfizer [3], reporting trough MPA concentrations of 0.80, 0.79, and 1.03 ng/mL, respectively, after 6, 12, and 24 months of DMPA-IM use (results from this study were included in the NDA for Pfizer's 104 mg subcutaneous product).

Predicted MPA concentrations 3 to 9 months after stopping DMPA-IM use under the above assumptions about apparent half-life, trough MPA levels, and variability are presented in Figure S1. Results are presented separately for women who receive only a single injection prior to stopping and for women who had achieved steady state after one year. Predicted mean MPA concentrations six months after last injection are 0.16 ng/mL and 0.22 ng/mL, respectively, in those two exposure groups. Approximately 95% of women who had achieved steady state are expected to have MPA concentrations below 0.4 ng/mL at 6-months after last DMPA-IM injection, but essentially all would have detectable MPA (>0.02 ng/mL) at that time point.

When assuming a 3-month trough of 0.58 ng/mL following initiation of DMPA-IM (the BE study result) and a half-life of 50 days (from Pfizer prescribing information), the predicted trough is approximately 0.80 ng/mL after 4 injection cycles (the observed value in [3]). Hence the data sources used to inform the modeling assumptions are consistent with one-another. A sensitivity analysis was also performed when assuming a 3-month trough of only 0.5 ng/mL and a half-life of only 30 days. Under these assumptions, 95% of women would be expected to have detectable MPA six months after last DMPA-IM injection (results not shown).

Based on these considerations, we assumed that women with MPA concentrations exceeding 0.4 ng/mL in their baseline specimens were likely to have used DMPA-IM in the previous 6-months, contrary to the study inclusion criteria regarding recent use of any of the study contraceptives.

Predicted serum MPA concentrations 90 to 270 days after a final injection of DMPA-IM among women who had achieved steady state after one year of use (red) or who received only a single injection before stopping (black). Shaded region contains 5th to 95th percentiles for steady-state group*

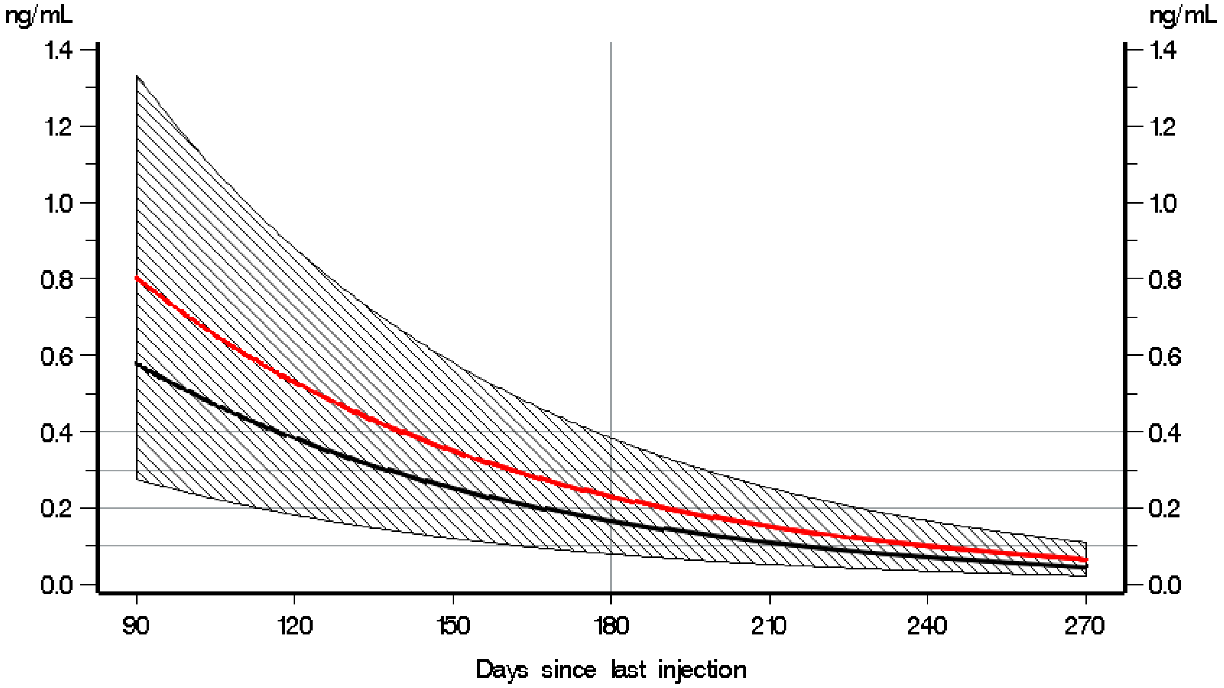

* Assumptions include: single-dose trough of 0.58 n/mL, apparent half-life of 50 days, terminal phase of absorption achieved by day 90, and a coefficient of variation of 40%.

References

1. U.S. FDA. Medroxyprogesterone acetate injectable suspension, 150 mg/mL prefilled syringe ANDA no.: 76–553. Center for Drug Evaluation and Research. Bioequivalence review. http://www.accessdata.fda.gov/drugsatfda_docs/nda/2004/076553_S000_Medroxyprogesterone_BIOPHARMR.pdf.
2. *Depo Provera CI (medroxyprogesterone acetate) injectable suspension 150 mg/1 mL [prescribing information]*. Revised October 2010, Pharmacia and Upjohn, Division of Pfizer Inc.: New York, NY.
3. Kaunitz AM., et al., *Subcutaneous DMPA vs. intramuscular DMPA: a 2-year randomized study of contraceptive efficacy and bone mineral density.* Contraception, 2009. **80**(1): p. 7-17.

Figure S2. HIV testing algorithm

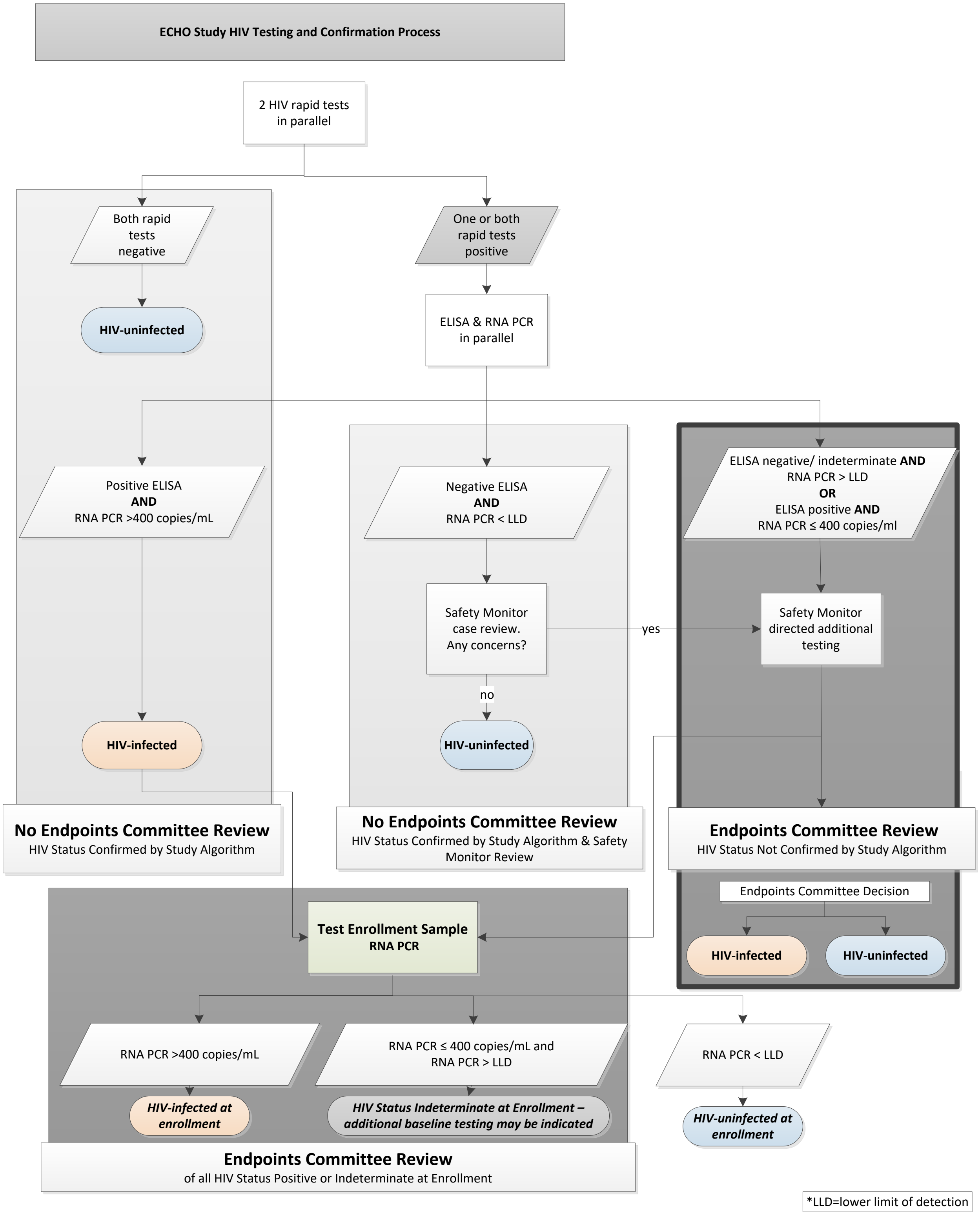

Figure S3. Pregnancy adjudication algorithm

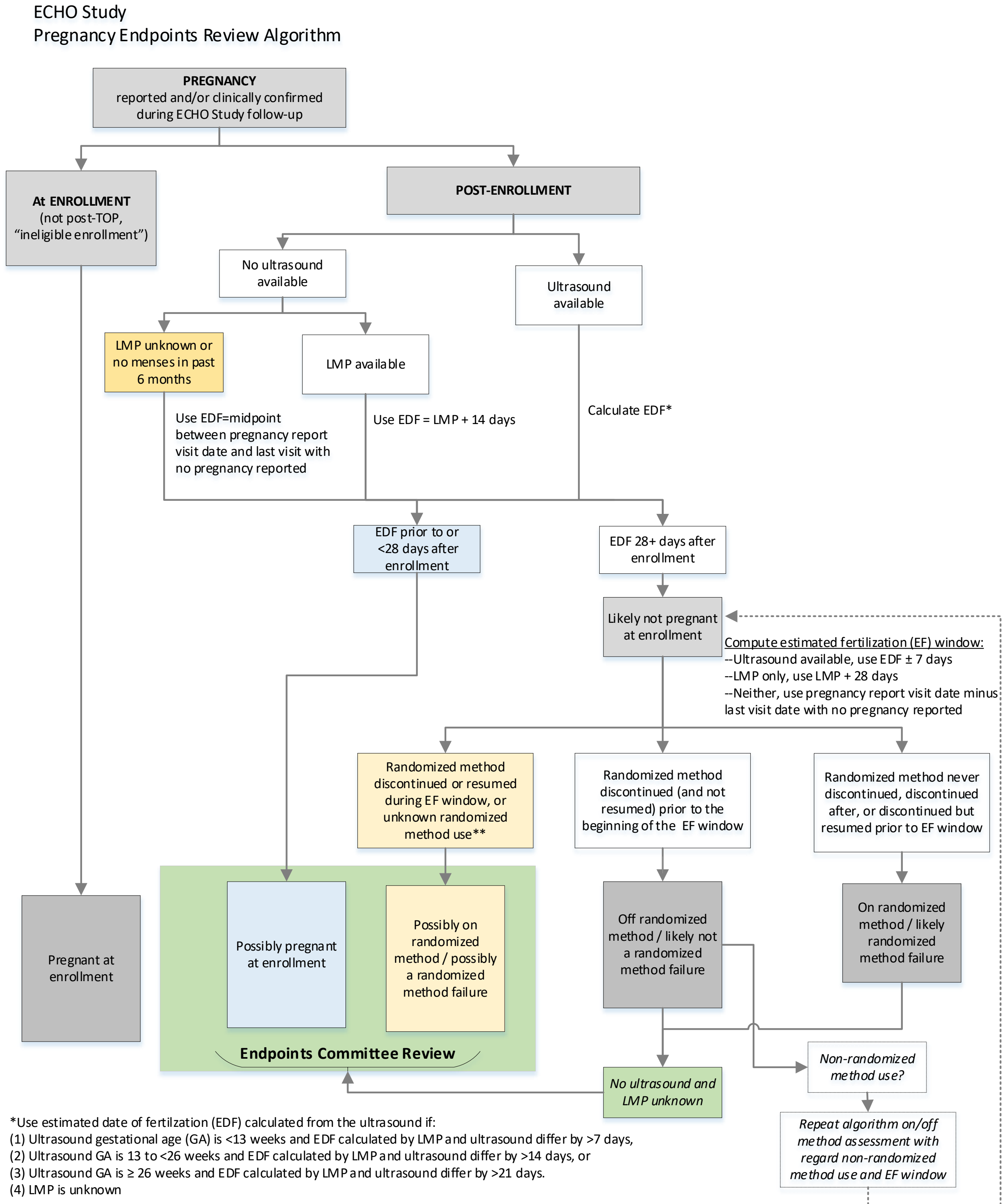

*Use estimated date of fertilzation (EDF) calculated from the ultrasound if:
(1) Ultrasound gestational age (GA) is <13 weeks and EDF calculated by LMP and ultrasound differ by >7 days,
(2) Ultrasound GA is 13 to <26 weeks and EDF calculated by LMP and ultrasound differ by >14 days, or
(3) Ultrasound GA is ≥ 26 weeks and EDF calculated by LMP and ultrasound differ by >21 days.
(4) LMP is unknown

EDF by LMP = LMP + 14 days
EDF by Ultrasound = (date of ultrasound – gestational age by ultrasound) +14 days

**Partially unknown / imputed randomized method discontinuation date (e.g., unknown date of discontinuation per participant recall / pending clinic records, and unknown IUD expulsions later confirmed by ultrasound)

Figure S4. Enrolment, retention, and outcomes

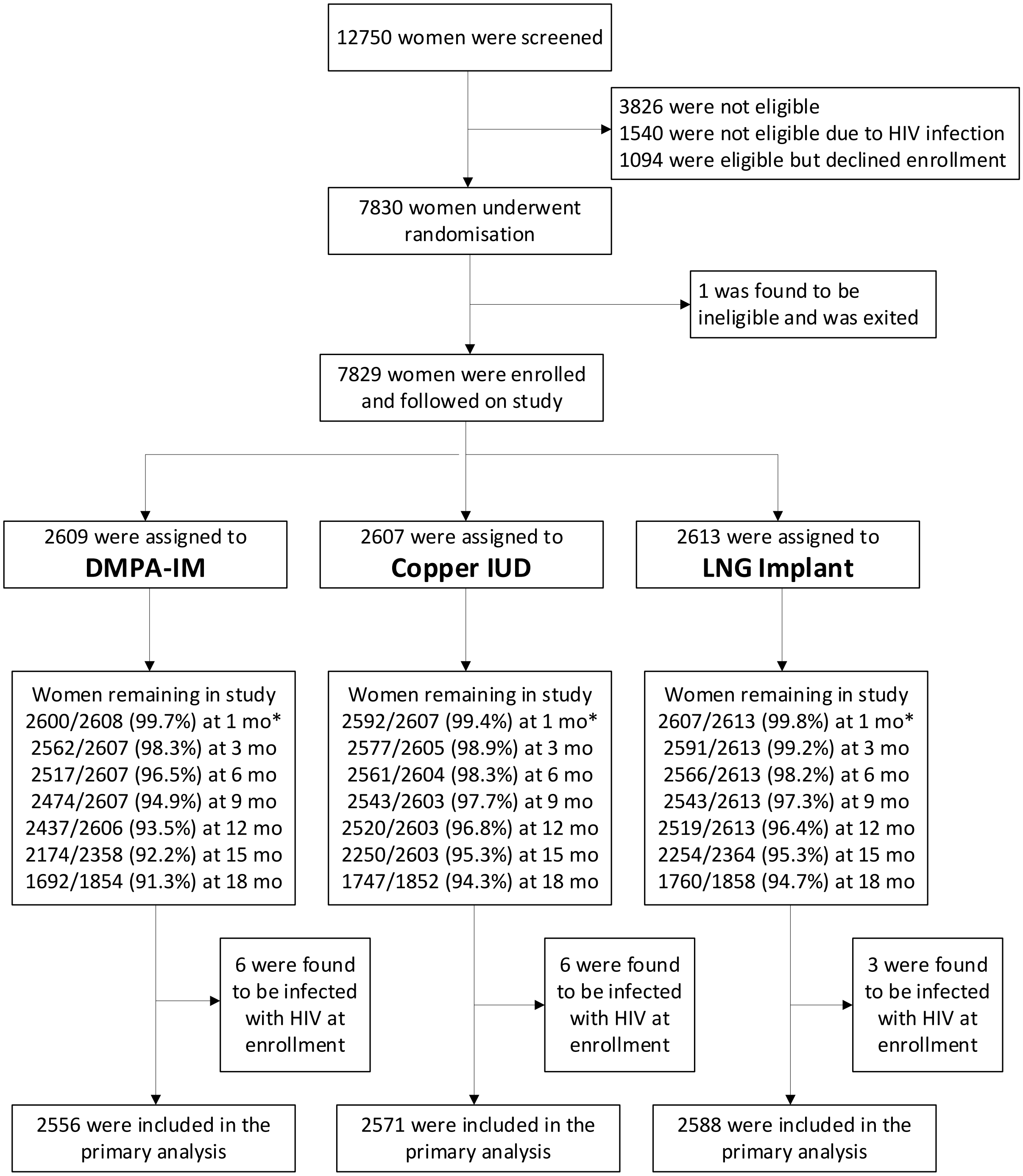

*Not an HIV-testing visit

Women remaining in study (3 mo – 18 mo): For each month, women are included in the denominator if they had not died prior to the visit window opening or completing the visit for that visit month, and if they were expected to attend the visit based upon expected exit at 12 mo, 15 mo, or 18 mo. Women are included in the numerator if they had an HIV test at that visit month or at a later visit (i.e., they were retained beyond the visit month). Seroconverters are counted in the numerator and denominator until planned study exit.

Women remaining in study (1 mo): The same definition as above was used for the 1 mo visit, with the exception that attendance of the visit (not HIV testing) was required for inclusion in the numerator.
